# Polyparasitism in Two-Stranded Dolphins: Multisystem Pathological Alterations and Cellular Responses

**DOI:** 10.3390/ani16172808

**Published:** 2026-09-07

**Authors:** Patcharaporn Kaewmong, Piyarat Khumraksa, Tatsawan Suttiboon, Nuttapong Limusunno, Domechai Kaewnoi, Sasibha Jantrakajorn, Peerapon Sornying, Narissara Keawchana, Pornphutthachat Sota, Pimwarang Sukkarun, Apinya Arnuphapprasert, Watcharapol Suyapoh

**Affiliations:** 1 Phuket Marine Biological Center, Wichit, Phuket 83000, Thailand; marineanimal.vet@gmail.com; 2 Marine and Coastal Resources Research Center (Lower Andaman Sea), Trang 92150, Thailand; piyarat.lmbc@gmail.com (P.K.); tatsawanunderthesea@gmail.com (T.S.); nuttapong.lim333@gmail.com (N.L.); 3 Department of Veterinary Science, Faculty of Veterinary Science, Prince of Songkla University, Songkhla 90110, Thailand; domechai.k@psu.ac.th (D.K.); sasibha.j@psu.ac.th (S.J.); peerapon.s@psu.ac.th (P.S.); narissara.k@psu.ac.th (N.K.); 4 School of Animal Technology and Innovation, Institute of Agricultural Technology, Suranaree University of Technology, Nakhon Ratchasima 30000, Thailand; pptc.sota@gmail.com; 5 Faculty of Veterinary Science, Rajamangala University of Technology Srivijaya, Nakhon Si Thammarat 80240, Thailand; pimwarang.s@gmail.com (P.S.); apinya.a@rmutsv.ac.th (A.A.); 6 Dugong and Seagrass Research Station, Prince of Songkla University, Songkhla 90110, Thailand

**Keywords:** *Halocercus delphini*, *Clistobothrium*, *Tetrabothrius*, polyparasitism, cetacean pathology

## Abstract

Polyparasitism is frequently documented in cetaceans; however, the pathological consequences of concurrent multispecies helminth infections and their associated tissue responses remain incompletely understood. This study characterized multispecies helminth infections in two live-stranded dolphins from the Andaman coast of Thailand, a spinner dolphin (*Stenella longirostris*) and a pantropical spotted dolphin (*Stenella attenuata*), using integrated postmortem, parasitological, molecular, histopathological, and immunohistochemical approaches. Both dolphins harbored helminths involving multiple organ systems. Pulmonary nematodes were identified as *Halocercus delphini*, while larval and intestinal cestodes were identified as *Clistobothrium* sp. and *Tetrabothrius* sp., respectively. The most prominent pathological alterations occurred in the respiratory tract and included chronic pyogranulomatous bronchopneumonia, airway epithelial injury, fibrosis, and pulmonary congestion associated with *Halocercus* infection. Additional parasite-associated lesions included chronic enteritis, cholangitis with biliary epithelial injury and ductular reaction, pancreatic inflammation, and localized inflammatory responses surrounding larval cestode cysts in the blubber and testis. Immunohistochemistry demonstrated extensive interleukin-4 (IL-4) immunoreactivity in affected pulmonary and intestinal tissues, whereas interleukin-10 (IL-10) was undetectable. Nitrotyrosine and heat shock protein 70 (HSP70) immunoreactivity were widely distributed across parasite-associated tissues, while caspase-3 immunoreactivity showed tissue-dependent patterns. Collectively, these findings demonstrate that multispecies helminth infections in stranded dolphins were associated with multisystem pathological alterations accompanied by inflammatory, oxidative/nitrosative stress, cellular stress, and apoptotic responses. These findings highlight the potential pathological significance of polyparasitism in stranded dolphins and support consideration of parasite burden and associated tissue responses during cetacean postmortem investigations.

## Simple Summary

Parasitic worms are commonly found in dolphins, but little is known about how simultaneous infections with several types of worms affect different organs within the same animal. This study investigated two dolphins stranded along the Andaman Sea coast of Thailand to determine the types and distribution of parasitic worms and the tissue changes associated with their infections. Both dolphins were infected with more than one type of parasitic worm, involving several organs, including the respiratory and digestive systems, liver, blubber, and reproductive tissues. The most substantial tissue damage occurred in the lungs, whereas parasites in other organs generally caused more localized inflammation and tissue injury. Examination of affected tissues also showed changes associated with inflammation, cellular stress, tissue-damaging reactive molecules, and programmed cell death. Together, these findings show that infection with multiple types of parasitic worms can be associated with widespread pathological changes rather than being limited to a single organ. Although these infections cannot be established as the cause of stranding or death in these two dolphins, recognizing their potential health effects may improve postmortem investigations, disease surveillance, and health assessment of stranded dolphins.

## 1. Introduction

Small cetaceans, including dolphins and porpoises, play important ecological roles in marine ecosystems through their contributions to biodiversity, trophic interactions, nutrient cycling, and community dynamics [1]. Cetacean strandings occur worldwide and may result from a complex combination of environmental, biological, and anthropogenic factors [2]. Proposed factors associated with stranding events include navigational disturbances related to bathymetric features, coastal configurations, or geomagnetic anomalies [3,4], climate or oceanographic events [5,6], anthropogenic noise and sonar interference [7,8], pollution [9], behavioral pattern [10], and infectious diseases, particularly those caused by bacteria, viruses, protozoa, and parasitic helminths [11,12].

Polyparasitism generally refers to the concurrent infection of an individual host with multiple parasite species or taxa [13]. The term encompasses concurrent infections regardless of whether each parasite taxon is associated with clinically significant disease, as the pathological consequences may vary considerably among co-infecting parasites and affected tissues. Helminth infections are common among marine mammals and encompass a diverse range of nematodes, cestodes, trematodes, and acanthocephalans [14,15]. In spinner dolphins (*Stenella longirostris*) and pantropical spotted dolphins (*Stenella attenuata*), several helminth taxa have been documented across different geographical regions, including Mexico, the Mediterranean Sea, the eastern tropical Pacific, and the Andaman Sea of Thailand. Reported parasites include digenean trematodes, tetrabothriid and phyllobothriid cestodes, polymorphid acanthocephalans, anisakid nematodes, and pseudaliid lungworms [16,17,18,19]. Although some infections may remain clinically inapparent, heavy or extensive parasitic infections have been associated with tissue injury, impaired organ function, debilitation, and, in severe cases, mortality in cetaceans [20,21].

The pathological consequences of helminth infection vary considerably according to parasite species, developmental stage, anatomical location, infection intensity, and host response [22,23]. Tissue alterations may arise through several mechanisms, including mechanical injury caused by parasite attachment, migration, feeding, or physical obstruction; local inflammatory and immune responses to adult parasites, larvae, eggs, or degenerating parasitic material; parasite-associated tissue necrosis and fibrosis; and secondary microbial infection of damaged tissues [24,25,26,27,28]. Chronic helminth-associated inflammation may additionally be accompanied by the generation of reactive oxygen and nitrogen species and cellular stress responses, potentially contributing to progressive tissue injury. Previous pathological investigations of cetaceans have documented parasite-associated inflammatory lesions in several organ systems, particularly the respiratory, gastrointestinal, hepatobiliary, and subcutaneous tissues [29,30,31]. However, most pathological descriptions of cetacean helminthiasis have focused on individual parasite taxa or lesions within specific organs. The pathological significance of concurrent multispecies helminth infections (polyparasitism) and the biological responses occurring across multiple affected tissues remain less well characterized. In particular, the relationships among parasite-associated inflammation, oxidative/nitrosative stress, cellular stress, and apoptosis in naturally infected dolphins have received limited investigation.

Therefore, this study characterized multispecies helminth infections in two live-stranded dolphins, a spinner dolphin (*S. longirostris*) and a pantropical spotted dolphin (*S. attenuata*), from the Andaman coast of Thailand. An integrated approach combining postmortem examination, morphological and molecular parasite identification, histopathology, and immunohistochemistry was used to characterize the anatomical distribution of helminths, associated tissue alterations, and local host responses. Accordingly, this study aimed to characterize multispecies helminth infections and determine whether parasite-associated lesions were accompanied by inflammatory, oxidative/nitrosative stress, cellular stress, and apoptotic responses in affected tissues. This study provides a detailed pathological characterization of polyparasitism in stranded dolphins and contributes to understanding its potential health significance in naturally infected small cetaceans.

## 2. Materials and Methods

### 2.1. Animal History and Study Design

In August 2023, two live-stranded dolphins were reported at separate locations within Maifad Subdistrict, Sikao District, Trang Province, Thailand. The first individual, a spinner dolphin (*S*. *longirostris*), designated TRCE076, was found at 7°20′37.3″ N, 99°22′03.7″ E. The second individual, a pantropical spotted dolphin (*S*. *attenuata*), designated TRCE078, was found at 7°29′59.4″ N, 99°19′24.0″ E. The geographic coordinates of both stranding sites were recorded and mapped using ArcGIS Pro version 2.8.0 (ESRI Inc., Redlands, CA, USA).

**Figure 1:**
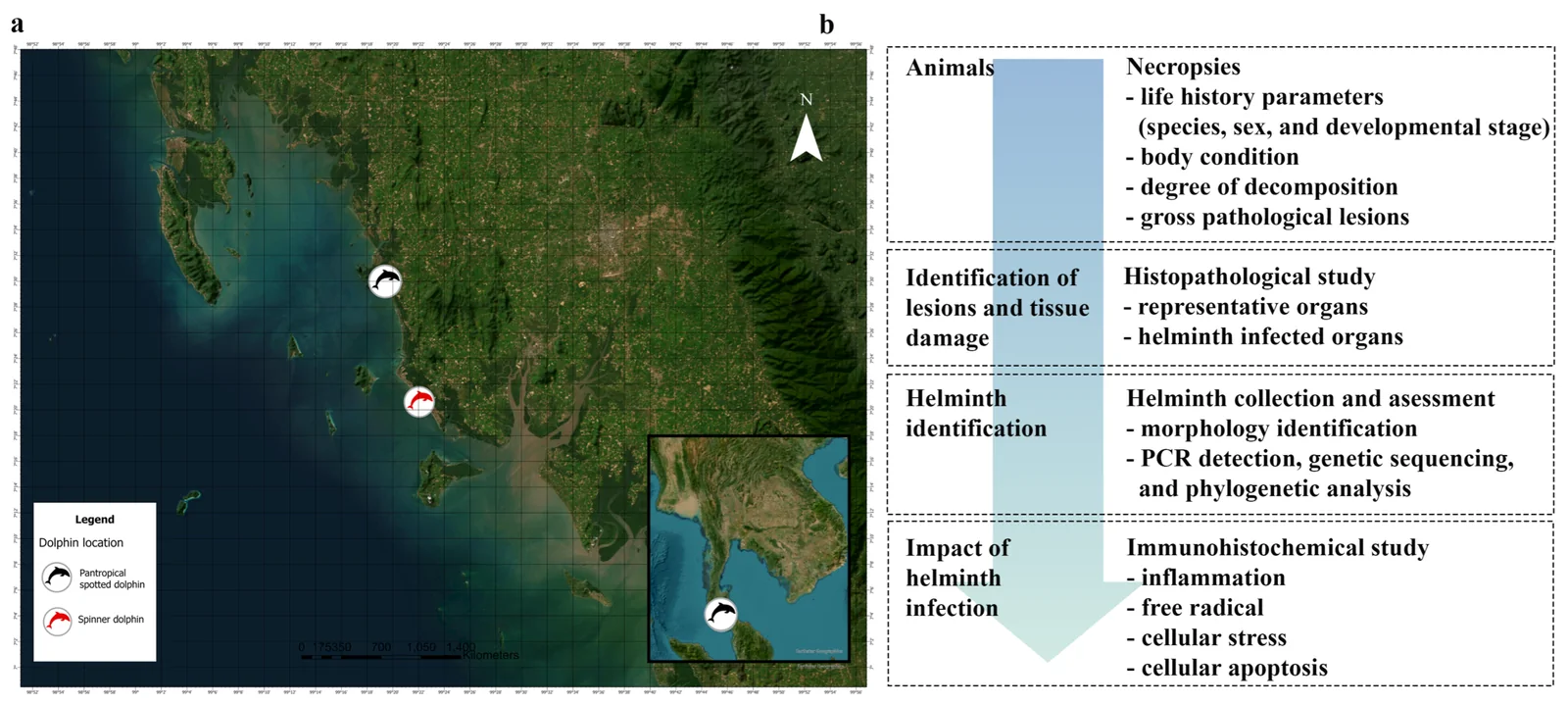
Geographic locations of two live-stranded dolphins and schematic overview of the study design. (**a**) Map showing the stranding locations of the spinner dolphin (*Stenella longirostris*; TRCE076) and pantropical spotted dolphin (*Stenella attenuata*; TRCE078) in Maifad Subdistrict, Sikao District, Trang Province, Thailand. (**b**) Schematic overview of the study framework used to characterize multispecies helminth infections and their associated pathological responses. Following postmortem examination, helminths were subjected to morphological and molecular identification, while affected tissues were evaluated by histopathology and immunohistochemistry to characterize inflammatory, oxidative/nitrosative stress, cellular stress, and apoptotic responses.

Following stranding, both dolphins were transported to the Marine and Coastal Resources Research Center (Lower Andaman Sea) for clinical assessment and supportive care; however, both animals died on the day of stranding. The study subsequently integrated complete postmortem examination, morphological and molecular identification of recovered helminths, histopathological evaluation of parasite-associated tissue lesions, and immunohistochemical characterization of inflammatory, oxidative/nitrosative stress, cellular stress, and apoptotic responses (Figure 1).

### 2.2. Postmortem Examination and Sample Collection

Complete postmortem examinations were performed according to standardized marine mammal necropsy procedures [32]. Prior to necropsy, species, sex, developmental stage, body condition, and decomposition status were recorded. External examination was performed systematically to identify traumatic injuries, cutaneous lesions, external parasites, and other gross abnormalities. The thoracic and abdominal cavities were subsequently opened, and all major organ systems were examined systematically. The anatomical distribution, extent, and characteristics of gross lesions were recorded and photographed.

Particular attention was given to the presence and anatomical distribution of helminths and their associated gross pathological lesions. The respiratory tract, including the trachea, primary and distal bronchi, and pulmonary parenchyma, was examined for pulmonary nematodes. The gastrointestinal tract was opened longitudinally and examined for luminal helminths and associated mucosal alterations. The liver and biliary tract, pancreas, blubber, and reproductive organs were similarly examined for adult helminths, larval stages, parasitic cysts, and associated tissue alterations. Representative helminths were collected directly from affected organs for subsequent morphological and molecular identification.

Representative tissue samples were collected from gross lesions and major organs, including the trachea, lung, heart, liver, spleen, kidney, stomach, intestine, pancreas, brain, blubber, and testis, when available. Samples intended for histopathological and immunohistochemical examination were fixed in 10% neutral buffered formalin. Representative helminths intended for morphological examination were preserved in 10% neutral buffered formalin, whereas specimens selected for molecular analysis were preserved in absolute ethanol until DNA extraction.

### 2.3. Morphological Identification of Helminths

Helminths collected during necropsy were initially categorized according to their anatomical location and gross morphological characteristics. Representative specimens were subsequently subjected to morphological examination using conventional carmine staining [33]. Formalin-fixed helminths were rinsed twice in tap water and immersed in carmine solution for 2–6 h. Excess stain was removed by differentiation in acid alcohol consisting of 2 mL concentrated HCl in 100 mL of 70% ethanol for 20 s to 2 min. Specimens were subsequently dehydrated through a graded ethanol series (50%, 70%, 85%, 96%, and 100%; 15 min each), cleared in alcohol–xylene for 30 min followed by xylene for 30 min, and prepared for microscopic examination.

Pulmonary nematodes were morphologically evaluated based on general body configuration and taxonomically informative anatomical structures, including the anterior end, lips, pharynx, intestinal tract, and reproductive organs. Female nematodes were examined for the ovary, uterus, eggs, and intrauterine larvae, whereas male nematodes were evaluated for the testes and copulatory spicules. Adult cestodes recovered from the intestinal lumen were examined for scolex morphology, suckers, neck, strobilar segmentation, and the development of immature, mature, and gravid proglottids. Reproductive structures, including the testes, sperm duct, genital pore, ovary, and uterus, were additionally evaluated. These morphological characteristics were used for preliminary identification of the pulmonary nematodes and adult intestinal cestodes.

Larval cestodes recovered from tissue cysts were examined for gross and microscopic characteristics of the larval body, scolex, and associated cystic structures. Because species-level identification of larval cestodes based solely on morphology is limited, morphological findings were interpreted together with subsequent molecular characterization using partial 18S rRNA sequences.

Morphological examination included 14 adult pulmonary nematodes (eight males and six females), six adult intestinal cestodes, four larval cestode specimens recovered from the testis, and three larval cestode specimens recovered from the liver. Specimens were examined using a Nikon ECLIPSE Ni-U upright microscope equipped with a digital imaging system (Nikon, Tokyo, Japan). Morphological features were photographed and documented using NIS-Elements Imaging Software, version 6.10 (Nikon, Tokyo, Japan), and representative anatomical characteristics were illustrated for taxonomic comparison.

### 2.4. Molecular Identification and DNA Sequencing

Genomic DNA was extracted from eight representative helminth specimens collected from the two dolphins using the GeneJET Genomic DNA Purification Kit (Thermo Scientific™, Waltham, MA, USA). The specimens comprised two pulmonary nematodes, one from each dolphin (NPTRCE076 and NPTRCE078); five larval cestodes, including one from the liver (CLTRCE076), two from the blubber (USKTRCE076 and CystTRCE078), and two from the testes (UTTRCE076 and UTTRCE078); and one adult intestinal cestode (CITRCE076). The second internal transcribed spacer (ITS2) of nuclear ribosomal DNA was selected to be the barcoding genetic marker for pseudaliid nematode identification due to its reliability in nematode species identification [34]. The primers PseuITS2F (5′-CCT TCG GCA CAT CTT GTT CA-3′) and PseuITS2R (5′-GGG TAA TCA CAT CTG AGT TCA-3′) [35] were used at a concentration of 10 pmol/µL. PCR reaction consisted of a final volume of 25 µL containing 25–40 ng of DNA template, 0.5 µL of each forward and reverse primer, 12.5 µL of OnePCR Ultra Master Mix (Bio-Helix, New Taipei City, Taiwan), and 5.5 µL of nuclease-free water. The PCR mixtures were amplified in a PCR thermal cycler (Eppendorf, Hamburg, Germany) under the following conditions: an initial denaturation at 94 °C for 3 min, 39 cycles consisting of denaturation at 94 °C for 1 min, annealing for 1 min at 51 °C, extension for 1 min at 72 °C, and a final extension at 72 °C for 10 min.

For the molecular identification of cestodes, 18S rRNA gene sequencing was chosen with the primers Ces1 (5′-CCA GCA GCC GCG GTA ACT CCA-3′) and Ces2 (5′-CCC CCG CCT GTC TCT TTT GAT -3′) [36] at a concentration of 10 pmol/µL. PCR reaction mixtures had a final volume of 25 µL containing 25–40 ng of DNA template, 0.5 µL of each forward and reverse primer, 12.5 µL of OnePCR Ultra Master Mix (Bio-Helix), and 9.5 µL of nuclease-free water. After an initial denaturation at 95 °C for 15 min, the reaction consisted of 30 cycles of denaturation at 94 °C for 1 min, annealing at 60 °C for 1 min, and extension at 72 °C for 2 min, followed by final extension at 72 °C for 8 min and 68 °C for 15 min, according to the previously described protocol [36].

The amplified products from both PCR reactions were purified using a GenepHlow^TM^ Gel/PCR Kit (GenepHlow^TM^, Geneaid Biotech Ltd., New Taipei City, Taiwan) and sequenced using the Big Dye^TM^ Terminator v3.1 Cycle Sequencing Kit (Applied Biosystems, Foster City, CA, USA) on an Applied Biosystems 3730 DNA Analyzer (ATGC, Bangkok, Thailand). Nucleotide sequences from both strands were used to assemble consensus sequences with BioEdit V.7.2 software and subsequently compared with the sequences available in GenBank using the blastn tool of the National Centre for Biotechnology Information (NCBI; https://blast.ncbi.nlm.nih.gov/Blast.cgi (accessed on 14 January 2024)) to verify their nucleotide identity.

### 2.5. Phylogenetic Analysis

Three sets of sequences were aligned separately with MAFFT (https://www.ebi.ac.uk/Tools/msa/mafft/ (accessed on 1 December 2023)). Sequences of ITS2 obtained from lungworms in this study were aligned with sequences of the members of families Pseudaliidae and Filaroididae, while our sequences of 18S rRNA gene retrieved from phyllobothriid or tetrabothriid cestode were aligned with sequences of the members of families of Phyllobothriidae, Tetrabothriidae, Diphyllobothriidae, Rhinebothriidae, Cathetocephalidae, Disculicepitidae, Gastrolecithidae, Nippotaeniidae, Mesocestoididae, Onchobothriidae, and Dilepididae. Lungworm and cestode sequences were selected according to their availability on GenBank. Phylogenetic trees of the ITS2 and 18S rRNA sequences of helminths in this study were constructed using the neighbor-joining method [37] and a bootstrap test with 1000 replicates using MEGA11 software [38].

### 2.6. Evaluation of Histopathological Alterations

Representative samples from grossly affected and apparently unaffected tissues were fixed in 10% neutral buffered formalin and routinely processed for histopathological examination [39,40]. Tissues were dehydrated through graded ethanol, cleared in xylene, embedded in paraffin wax, sectioned at 4 μm, and stained with hematoxylin and eosin (H&E).

Histopathological examination was performed systematically on available tissues from the respiratory, gastrointestinal, hepatobiliary, pancreatic, subcutaneous, reproductive, cardiovascular, urinary, lymphoid, and nervous systems. Particular attention was given to lesions anatomically associated with adult helminths, larval stages, parasitic cysts, or eggs. For each affected tissue, lesions were characterized according to their anatomical distribution, extent, predominant inflammatory cell populations, and associated tissue alterations, including epithelial injury, necrosis, fibrosis, vascular changes, hemorrhage, congestion, and mineralization, where present.

Parasite-associated lesions were interpreted by integrating histological findings with the anatomical distribution of helminths observed during postmortem examination and their morphological and molecular identification. Histopathological findings in tissues without direct evidence of helminth infection were evaluated separately and were not attributed to helminth infection in the absence of a spatial association with parasitic structures.

### 2.7. Immunohistochemical Assessment of Host Responses Associated with Helminth Infection

Immunohistochemical (IHC) analyses were performed on formalin-fixed, paraffin-embedded sections of the lung, liver, intestine, blubber, and testis to characterize host responses associated with multispecies helminth infections. The selected markers included interleukin-4 (IL-4) and interleukin-10 (IL-10) for inflammatory and immunoregulatory responses, nitrotyrosine for oxidative/nitrosative stress, heat shock protein 70 (HSP70) for cellular stress, and caspase-3 for apoptosis. For all immunohistochemical analyses, intestinal tissue from a parasite-infected spotted scat (*Scatophagus argus*) previously confirmed to show immunoreactivity for IL-4, IL-10, nitrotyrosine, HSP70, and caspase-3 was used as the positive control. Negative controls were prepared by omitting the primary antibody while otherwise following the same immunohistochemical procedure. Non-infected tissues from healthy cetaceans were not available for use as biological baseline controls.

#### 2.7.1. Assessment of IL-4 and IL-10 Immunoreactivity

Immunohistochemical localization of IL-4 and IL-10 was performed according to the protocol described by Peng et al. (2022) [41], with appropriate modifications. Formalin-fixed, paraffin-embedded sections (4 μm) of the lung, liver, intestine, blubber, and testis were incubated at 60 °C for 15 min, deparaffinized in xylene, and rehydrated through a graded ethanol series. Antigen retrieval was performed in 10 mM citrate buffer (pH 6.0) using microwave heating at 100 W for 5 min followed by 20 W for 15 min. Sections were allowed to cool at room temperature for 30 min and subsequently washed twice with phosphate-buffered saline (PBS).

Endogenous peroxidase activity was quenched with 3% hydrogen peroxide in methanol, and nonspecific antibody binding was blocked by incubation with 1% bovine serum albumin (BSA) for 1 h at room temperature. Sections were subsequently incubated overnight at 4 °C with rabbit polyclonal anti-IL-4 (AF5142; Affinity Biosciences, Cincinnati, OH, USA) or rabbit polyclonal anti-IL-10 (DF6894; Affinity Biosciences, Cincinnati, OH, USA) primary antibodies, each diluted 1:500 in Tris-buffered saline (TBS).

The following day, sections were incubated for 1 h with a biotinylated goat anti-mouse/rabbit secondary antibody (K0675; DakoCytomation, Glostrup, Denmark) diluted 1:100. After three washes in PBS, a streptavidin–biotin–horseradish peroxidase complex (K0355; DakoCytomation; Glostrup, Denmark) was applied for 1 h. Immunoreactivity was visualized using 3,3′-diaminobenzidine (DAB) for 5 min. Sections were counterstained with Mayer’s hematoxylin, dehydrated through graded ethanol, cleared in xylene, and coverslipped for microscopic examination.

#### 2.7.2. Assessment of Oxidative/Nitrosative Stress

Nitrotyrosine immunohistochemistry was performed as an indicator of protein nitration associated with oxidative/nitrosative stress, following the protocol described by Cuzzocrea et al. (2000) [42], with modifications. Formalin-fixed, paraffin-embedded sections (4 μm) of the lung, liver, intestine, blubber, and testis were incubated at 60 °C for 15 min, deparaffinized in xylene, and rehydrated through a graded ethanol series.

Sections were permeabilized with 0.1% Triton X-100 in PBS for 20 min, followed by quenching of endogenous peroxidase activity with 5% hydrogen peroxide. Nonspecific binding was blocked by incubation with 1% BSA in PBS for 1 h at room temperature. Sections were subsequently incubated overnight at 4 °C with a rabbit polyclonal anti-nitrotyrosine antibody (A-21285; Thermo Fisher Scientific, Waltham, MA, USA) diluted 1:50 in PBS.

Immunoreactivity was visualized using the DAKO ChemMate EnVision Detection Kit (Dako, Glostrup, Denmark). Sections were counterstained with hematoxylin, dehydrated through graded ethanol, cleared in xylene, and mounted with a permanent mounting medium. Positive immunoreactivity was identified by brown DAB labeling within the relevant cellular and tissue compartments.

#### 2.7.3. Assessment of Cellular Stress and Apoptosis

HSP70 and caspase-3 were evaluated as markers of cellular stress and apoptosis, respectively, using immunohistochemical (IHC) localization. The IHC procedures were performed on 4 μm-thick paraffin-embedded sections of the lung, liver, intestine, blubber, and testis, following the protocols described by Ondruschka et al. (2018) [43] and Jantrakajorn et al. (2024) [44], with slight modifications. Antigen retrieval was conducted via microwave heating in 10 mM citric acid buffer (pH 6.0), initially at 100 W for 5 min and subsequently at 20 W for 15 min. Sections were then cooled for 30 min and rinsed twice with phosphate-buffered saline (PBS). Endogenous peroxidase activity was blocked by incubation with 3% hydrogen peroxide (H_2_O_2_) in methanol. To reduce nonspecific binding, the sections were incubated with 1% bovine serum albumin (BSA) for 1 h at room temperature. For HSP70 detection, a rabbit polyclonal anti-HSP70 antibody (SAB5700631; Sigma-Aldrich, St. Louis, MO, USA) was applied at a 1:500 dilution and incubated overnight. For the assessment of apoptosis, a rabbit polyclonal anti-caspase-3 antibody (SAB5700196; Sigma-Aldrich, St. Louis, MO, USA) was applied at a 1:200 dilution and incubated overnight. Immunoreactivity for both markers was visualized using the DAKO ChemMate Envision Detection Kit (Dako, Glostrup, Denmark). Finally, all sections were dehydrated through graded alcohols, cleared in xylene, and mounted with a permanent mounting medium. Positive immunostaining was identified by the presence of a brown precipitate—localized within the cytoplasm for HSP70, and within both the cytoplasm and nucleus for caspase-3.

#### 2.7.4. Semi-Quantitative Evaluation of Immunoreactivity

Immunoreactivity was evaluated semi-quantitatively according to previously described grading approaches for the respective immunohistochemical markers, including IL-4 and IL-10 [45], nitrotyrosine [40], HSP70 and caspase-3 [44,46]. For each marker, the entire tissue section was systematically examined rather than selected high-power fields. The proportion of positively immunoreactive cells was estimated across the whole section, with particular attention to parasite-associated lesions and the immediately surrounding tissues. Only areas with adequately preserved tissue morphology were included in the evaluation, whereas areas showing evident postmortem autolytic changes were excluded to minimize potential effects on the interpretation of immunoreactivity. Immunoreactivity was categorized according to the proportion of positively stained cells as follows: absent (−), ≤1%; weak (+), 2–25%; moderate (++), 26–50%; and strong (+++), >50%. In addition to the semi-quantitative grade, the predominant cellular and tissue localization of immunoreactivity was recorded for each marker, including inflammatory, epithelial, endothelial, stromal, interstitial, and parenchymal cells, as well as parasitic structures where applicable. The immunoreactivity scores and predominant staining patterns were subsequently summarized descriptively across the examined tissues.

## 3. Results

### 3.1. Clinical Characteristics of Stranded Dolphins

Two live-stranded male dolphins, a subadult spinner dolphin (*S. longirostris*; TRCE076) and an adult pantropical spotted dolphin (*S. attenuata*; TRCE078), were admitted to the Marine and Coastal Resources Research Center (Lower Andaman Sea), Thailand, in August 2023. Basic case characteristics and stranding information are summarized in Table 1. TRCE076 measured 178 cm in total body length, weighed 43.8 kg, and was relatively thin (BCS 2.5/5), whereas TRCE078 measured 200 cm and was in moderate body condition (BCS 3/5); body weight was not recorded for TRCE078.

Both dolphins were found alive and subsequently transported for clinical assessment and supportive care. Both presented with severe clinical deterioration and abnormal respiratory signs. Despite supportive care, both animals died shortly after stranding, and postmortem examinations were performed on fresh carcasses. Because of the rapid progression from stranding to death, ante-mortem hematological and serum biochemical examinations were not performed. Both carcasses subsequently underwent complete postmortem examination, parasitological investigation, molecular parasite identification, histopathological evaluation, and immunohistochemical analyses.

### 3.2. Postmortem and Parasitological Findings

#### 3.2.1. Gross Distribution of Helminths

Necropsy revealed a multispecies helminth burden involving multiple organ systems in both stranded dolphins (Table 2). The spinner dolphin (*S*. *longirostris*; TRCE076) exhibited broader parasite diversity and anatomical distribution, with helminths identified in the respiratory tract, gastrointestinal tract, liver, blubber, and testis. In comparison, the pantropical spotted dolphin (*S*. *attenuata*; TRCE078) harbored helminths in the respiratory tract, blubber, and testis.

**Figure 2:**
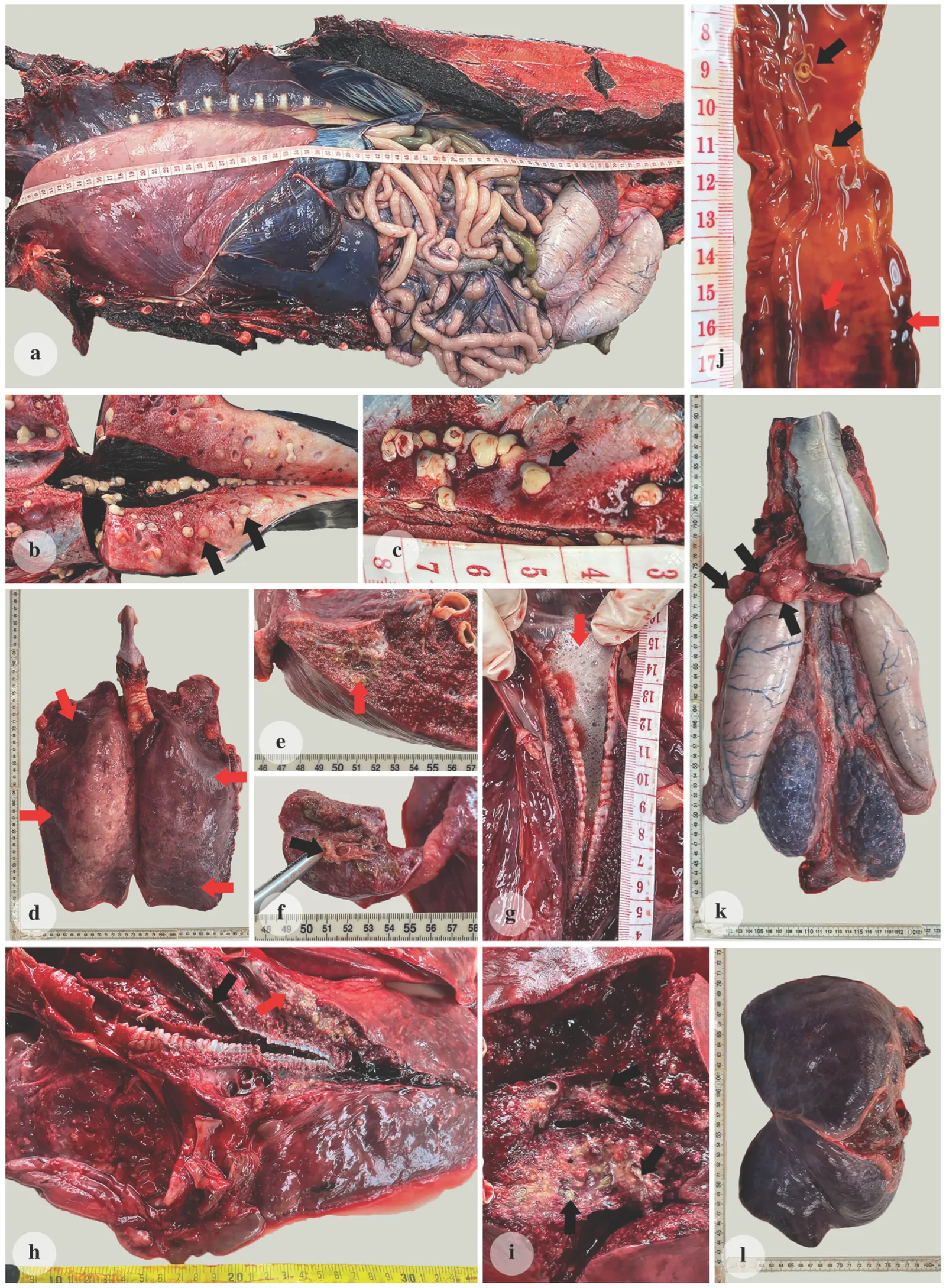
Gross pathological lesions associated with multispecies helminth burden in stranded dolphins. (**a**) Opening of the carcass during necropsy (TRCE076). (**b**) Multiple larval cestode cysts disseminated throughout the blubber (TRCE076). (**c**) Higher magnification of larval cestode cysts embedded within the blubber (TRCE076). (**d**) Lungs showing locally extensive consolidation involving the right lung lobe, whereas the left lung exhibited diffuse (panlobar) consolidation (TRCE076). (**e**) Cut surface of the lung demonstrating marked pulmonary consolidation and congestion (TRCE076). (**f**) Numerous slender white nematodes occupying the trachea, primary bronchi, and distal bronchi, associated with abundant frothy exudate and partial luminal obstruction (TRCE076). (**g**) Frothy exudate within the tracheal lumen (TRCE076). (**h**) Lung of TRCE078 showing numerous pulmonary nematodes within the tracheobronchial tree associated with diffuse pulmonary consolidation. (**i**) Cut surface of the lung showing extensive pulmonary consolidation with bronchi obstructed by numerous nematodes (TRCE078). (**j**) Adult cestodes within the intestinal lumen associated with mucosal congestion and hemorrhage (TRCE076). (**k**) Multiple larval cestode cysts distributed throughout the testicular tissue (TRCE076). (**l**) Liver exhibiting diffuse congestion with multifocal capsular pitting and mild capsular thickening suggestive of fibrosis (TRCE076). Black arrows indicate helminths, and red arrows indicate gross pathological lesions.

The respiratory tract exhibited the most severe gross pathological alterations in both dolphins. No external traumatic injuries or cutaneous lesions were identified. Upon opening the thoracic cavity, abundant serosanguineous fluid was present within the trachea and extended into the primary bronchi. Numerous slender white nematodes densely occupied the trachea, primary bronchi, and distal bronchi, where they were intermixed with abundant frothy to mucopurulent exudate, resulting in partial luminal obstruction (Figure 2f,g). The lungs exhibited diffuse congestion with multifocal to locally extensive areas of firm consolidation. In TRCE076, consolidation predominantly affected the right lung lobe, whereas the left lung showed diffuse (panlobar) involvement. On cut section, the pulmonary parenchyma displayed marked congestion and consolidation with multiple yellowish nodules scattered throughout the affected areas (Figure 2d,e). Similar pulmonary lesions were observed in TRCE078, in which numerous pulmonary nematodes occupied the tracheobronchial tree and obstructed bronchi within severely consolidated lungs (Figure 2h,i).

Outside the respiratory system, the heart was markedly congested, whereas the liver exhibited diffuse congestion with multifocal capsular pitting, mild capsular thickening, and a mottled appearance. The stomach was devoid of ingesta. In TRCE076, the intestinal lumen contained numerous adult cestodes associated with multifocal mucosal congestion and hemorrhage (Figure 2j). Multiple larval cestode cysts were disseminated throughout the blubber and testicular tissues of both dolphins and were additionally identified within the liver of TRCE076 (Figure 2b,c,k,l). Collectively, gross examination demonstrated a multispecies helminth burden involving multiple organ systems (Figure 2a).

#### 3.2.2. Morphological Characteristics of Identified Helminths

**Figure 3:**
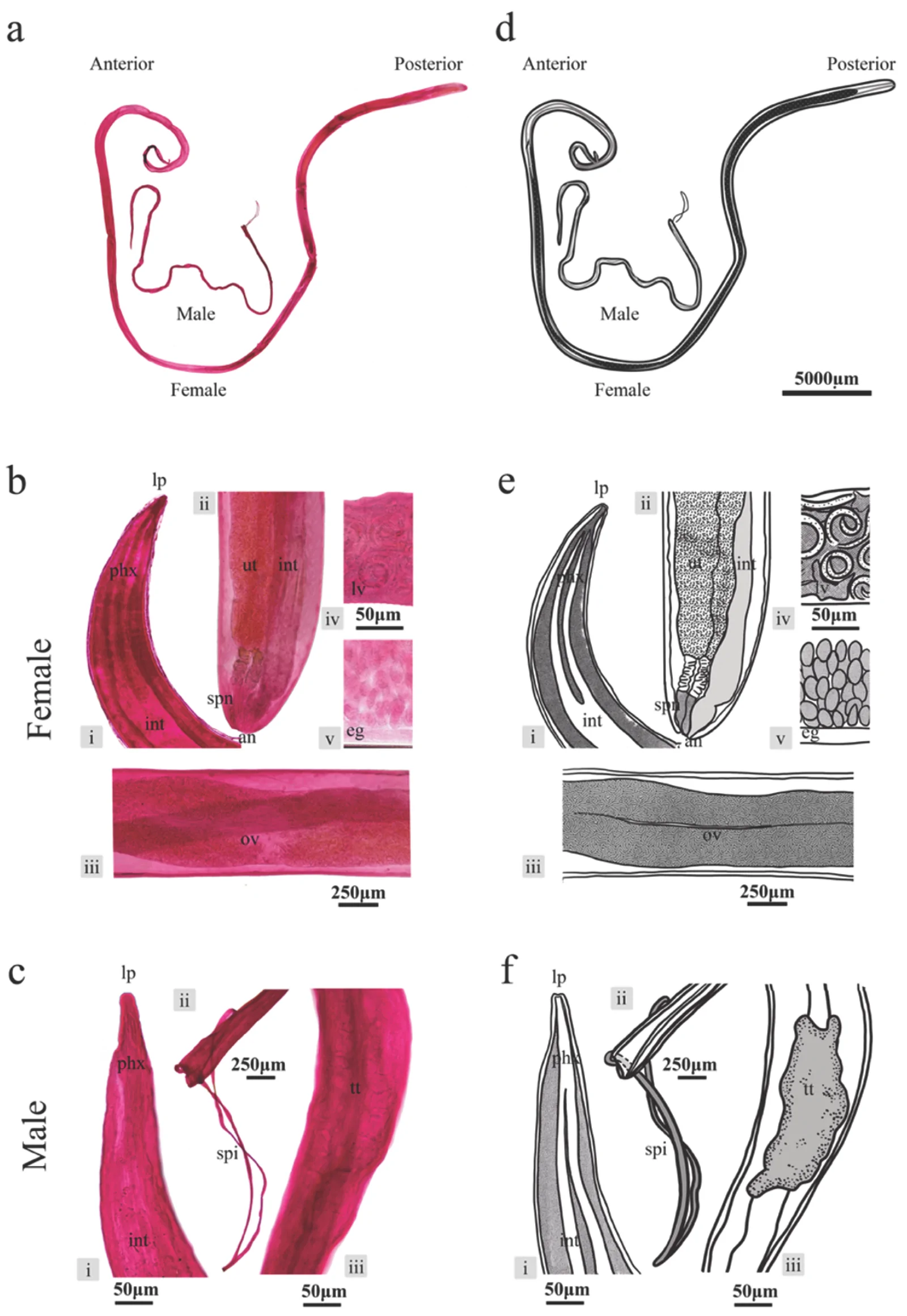
Representative photographs and drawings of *Halocercus* spp. showing major anatomical features. (**a**) Subgross of adult male and female nematodes. (**b**) Higher magnification of female structure. (**c**) Higher magnification of male structure. (**d**) Illustration of the subgross level. (**e**) Illustration of female structure. (**f**) Illustration of male structure. lp—Lips; phx—Pharynx; int—Intestine; ut—Uterus; spn—Muscular sphincter; an—Anus; eg—Egg; lv—Larva; ov—Ovary; spi—Spicule; tt—Testes. ((**a**–**c**) = carmine; (**d**–**f**) = Illustration, original magnification, (**a**,**d**) = subgross, scale bar depicts 5000 µm; (**bi**–**biii**,**ei**–**eiii**) = ×10, scale bar depicts 250 µm; (**biv**,**bv**,**eiv**,**ev**) = ×40, scale bar depicts 50 µm; (**cii**,**fii**) = ×4, scale bar depicts 250 µm; (**ci**,**ciii**,**fi**,**fiii**) = ×40, scale bar depicts 50 µm).

**Figure 4:**
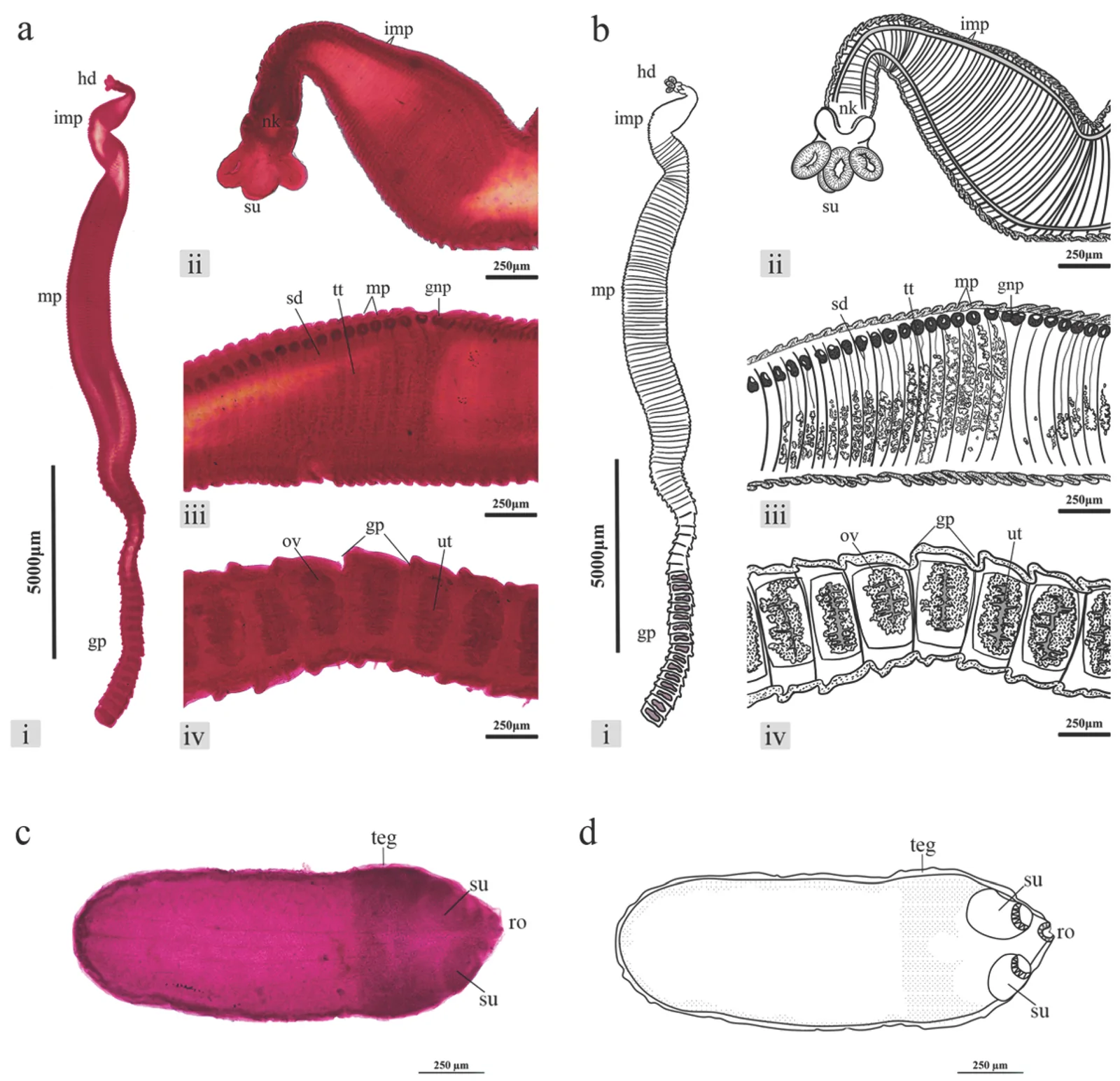
Representative photographs and drawings of *Tetrabothrius* spp. and *Clistobothrium* spp. showing major anatomical features. (**a**) Photomicrograph of an adult *Tetrabothrius* spp. (**b**) Illustration of adult *Tetrabothrius* spp. (**ai**,**bi**) Subgross appearance of *Tetrabothrius* spp. (**aii**,**bii**) Higher magnification of the head and immature proglottids. (**aiii**,**biii**) Higher magnification of mature proglottids. (**aiv**,**biv**) Higher magnification of reproductive structures within mature proglottids. (**c**) Photomicrograph of a *Clistobothrium* spp. plerocercoid. (**d**) Illustration of a *Clistobothrium* spp. plerocercoid. hd—head; imp—immature proglottids; mp—mature proglottids; gp—gravid proglottids; su—sucker; nk—neck; sd—sperm duct; tt—testes; gnp—genital pore; ov—ovary; ut—uterus; teg—tegument; ro—rostellum. (**a**,**c**) = carmine; (**b**,**d**) = illustration; original magnification: ((**ai**,**bi**) = subgross, scale bar = 5000 µm; (**aii**–**aiv**,**bii**–**biv**,**c**,**d**) = ×10, scale bar = 250 µm).

Morphological examination identified the pulmonary nematodes as *Halocercus* spp., the intestinal cestodes as *Tetrabothrius* spp., and the larval cestodes recovered from the liver, blubber, and testis as *Clistobothrium* spp. Representative morphological characteristics are shown in Figure 3 and Figure 4. Adult *Halocercus* spp. were slender, cylindrical nematodes with a distinct anterior end bearing lips surrounding the oral opening, followed by a muscular pharynx and a simple intestinal tract (Figure 3). Female worms contained paired ovaries, a well-developed uterus containing numerous eggs and larvae, and a prominent muscular sphincter near the posterior reproductive tract, whereas males possessed paired testes and well-developed copulatory spicules.

Adult intestinal cestodes were morphologically consistent with *Tetrabothrius* spp., characterized by a distinct scolex bearing four suckers, a short neck, and a segmented strobila composed of immature, mature, and gravid proglottids (Figure 4a,b). Mature proglottids contained well-developed male and female reproductive organs, including numerous testes, a sperm duct, genital pore, ovary, and uterus. Larval cestodes recovered from the liver, blubber, and testicular tissues were morphologically consistent with *Clistobothrium* spp., appearing as elongated, whitish plerocercoids with elongated to cystic bodies, a relatively thick tegument, and a small retractile rostellum at the anterior end of the scolex (Figure 4c,d).

#### 3.2.3. Molecular Identification and Phylogenetic Analyses

**Figure 5:**
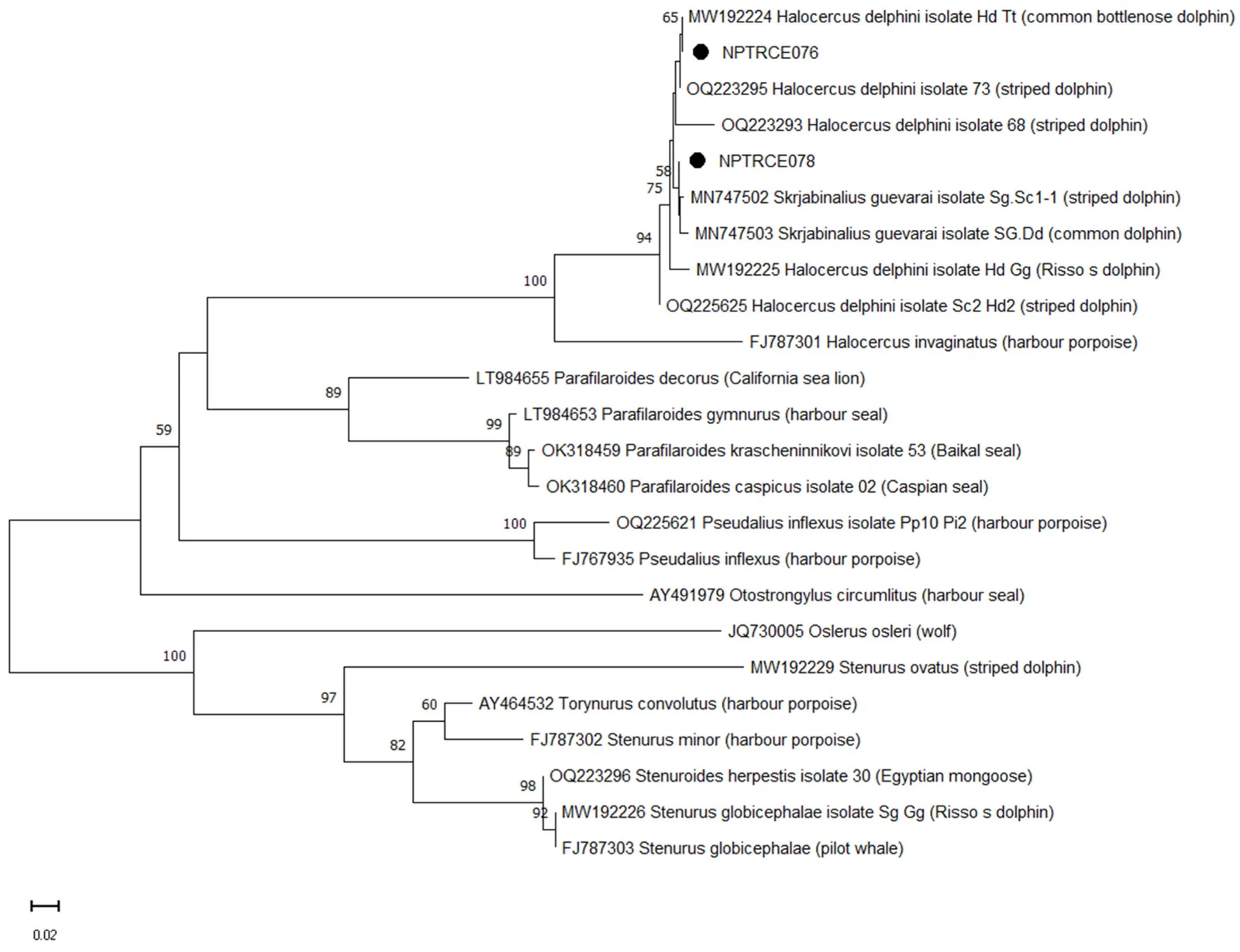
Neighbor-joining phylogenetic tree based on second internal transcribed spacer (ITS2) of nuclear ribosomal DNA of two pseudaliid lung nematodes infecting spinner dolphin and pantropical spotted dolphin in Thailand (marked with black circle), and other members of the Metastrongyloidea. Bootstrap percentages of >50% calculated from 1000 replications are displayed next to the branches. Scale bar represents 0.02 substitutions per nucleotide.

Molecular analyses confirmed the morphological identification of all helminths recovered from the stranded dolphins. The ITS2 sequences obtained from pulmonary nematodes (isolates NPTRCE076 and NPTRCE078) were approximately 480 bp in length and shared 98.66% nucleotide identity with each other. Both sequences exhibited 98.73–99.38% nucleotide identity with *Halocercus delphini* isolate Hd Tt 5.8S recovered from the common bottlenose dolphin (*Tursiops truncatus*) (GenBank accession no. MW192224). Phylogenetic analysis based on ITS2 sequences clustered both isolates within the *H. delphini* clade with strong support, clearly separating them from other pseudaliid lungworms, including *Stenurus* spp. and *Torynurus* spp. (Figure 5).

**Figure 6:**
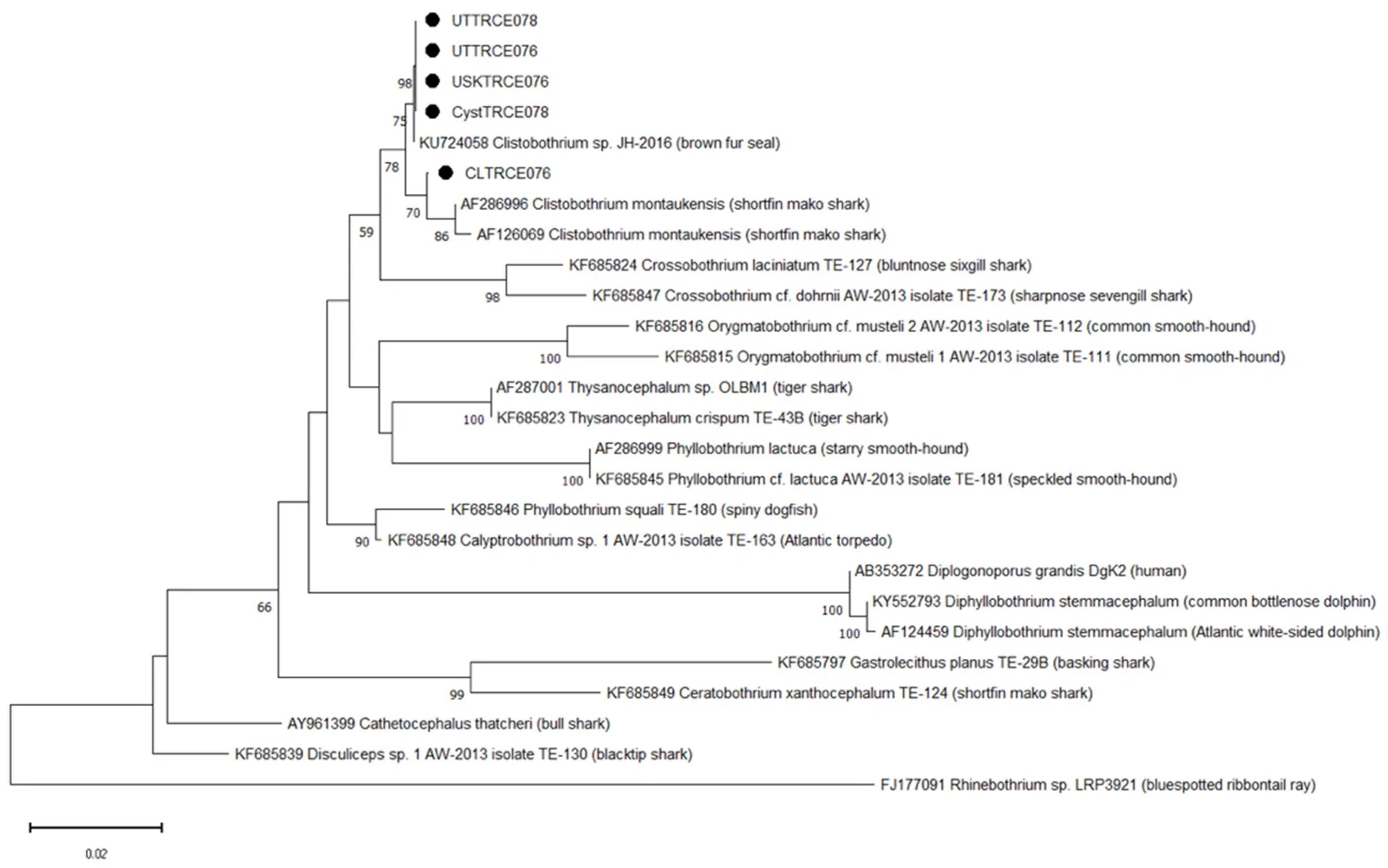
Neighbor-joining phylogenetic tree based on partial 18S rRNA sequences of five phyllobothriid cestodes infecting spinner dolphin and Pantropical spotted dolphin in Thailand (marked with black circle), and other cestodes. Bootstrap percentages of >50% calculated from 1000 replications are displayed next to the branches. Scale bar represents 0.02 substitutions per nucleotide.

Partial 18S rRNA sequences obtained from five larval cestodes recovered from different organs were approximately 430 bp in length and shared 99.53–99.54% nucleotide identity. All isolates exhibited 99.54% sequence similarity with *Clistobothrium* sp. JH-2016 isolated from the brown fur seal (GenBank accession no. KU724058) and *Clistobothrium montaukensis* isolated from the shortfin mako shark (GenBank accession no. AF286996). Phylogenetic analysis consistently grouped all isolates within the *Clistobothrium* clade irrespective of host species or anatomical location, with *Crossobothrium* spp. forming the sister lineage (Figure 6).

**Figure 7:**
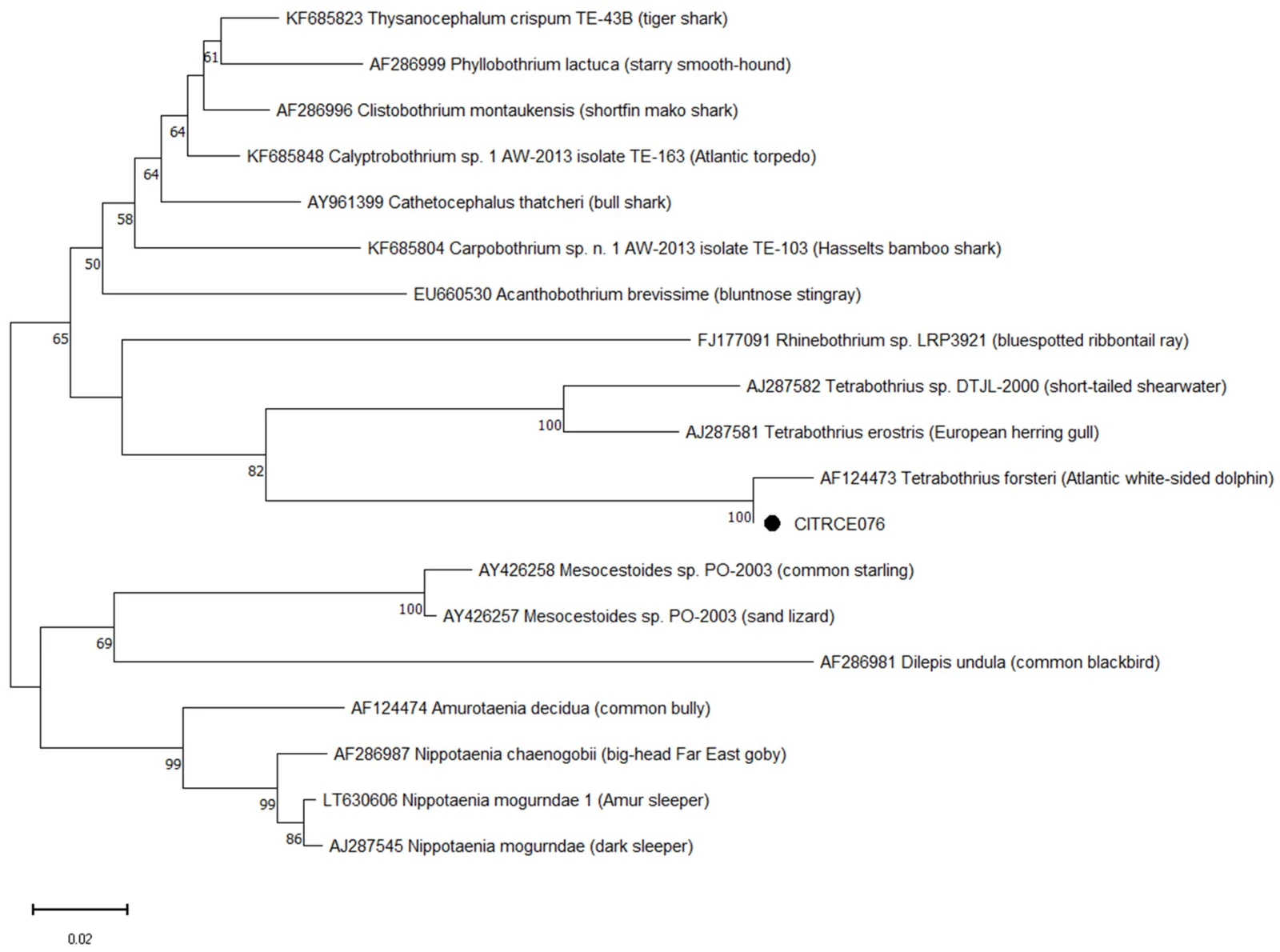
Neighbor-joining phylogenetic tree based on partial 18S rRNA sequences of the tetrabothriid cestode infecting spinner dolphin in Thailand (marked with black circle) and other cestodes. Bootstrap percentages of >50% calculated from 1000 replications are displayed next to the branches. Scale bar represents 0.02 substitutions per nucleotide.

The intestinal cestode isolate (CITRCE076) yielded an approximately 480 bp partial 18S rRNA sequence and was identified as *Tetrabothrius* sp., showing 94.67% nucleotide similarity to *Tetrabothrius forsteri* (GenBank accession no. AF124473). Phylogenetic reconstruction placed the isolate within the *Tetrabothrius* clade, closely related to *T. forsteri* and clearly distinct from *T. erostris* recovered from aquatic birds (Figure 7).

The nucleotide sequences generated in this study were deposited in GenBank under accession numbers PP097909–PP097910 (*Halocercus delphini*), PP091040–PP091044 (*Clistobothrium* sp.), and PP099502 (*Tetrabothrius* sp.).

### 3.3. Histopathological Alterations Associated with Multispecies Helminth Infections

Histopathological examination demonstrated that tissue lesions closely corresponded with the anatomical distribution of helminths identified during necropsy. Lesions were predominantly confined to the respiratory, gastrointestinal, hepatobiliary, subcutaneous, and reproductive systems, whereas other organs exhibited only mild circulatory changes or postmortem autolysis without significant parasite-associated pathology.

#### 3.3.1. Respiratory System

**Figure 8:**
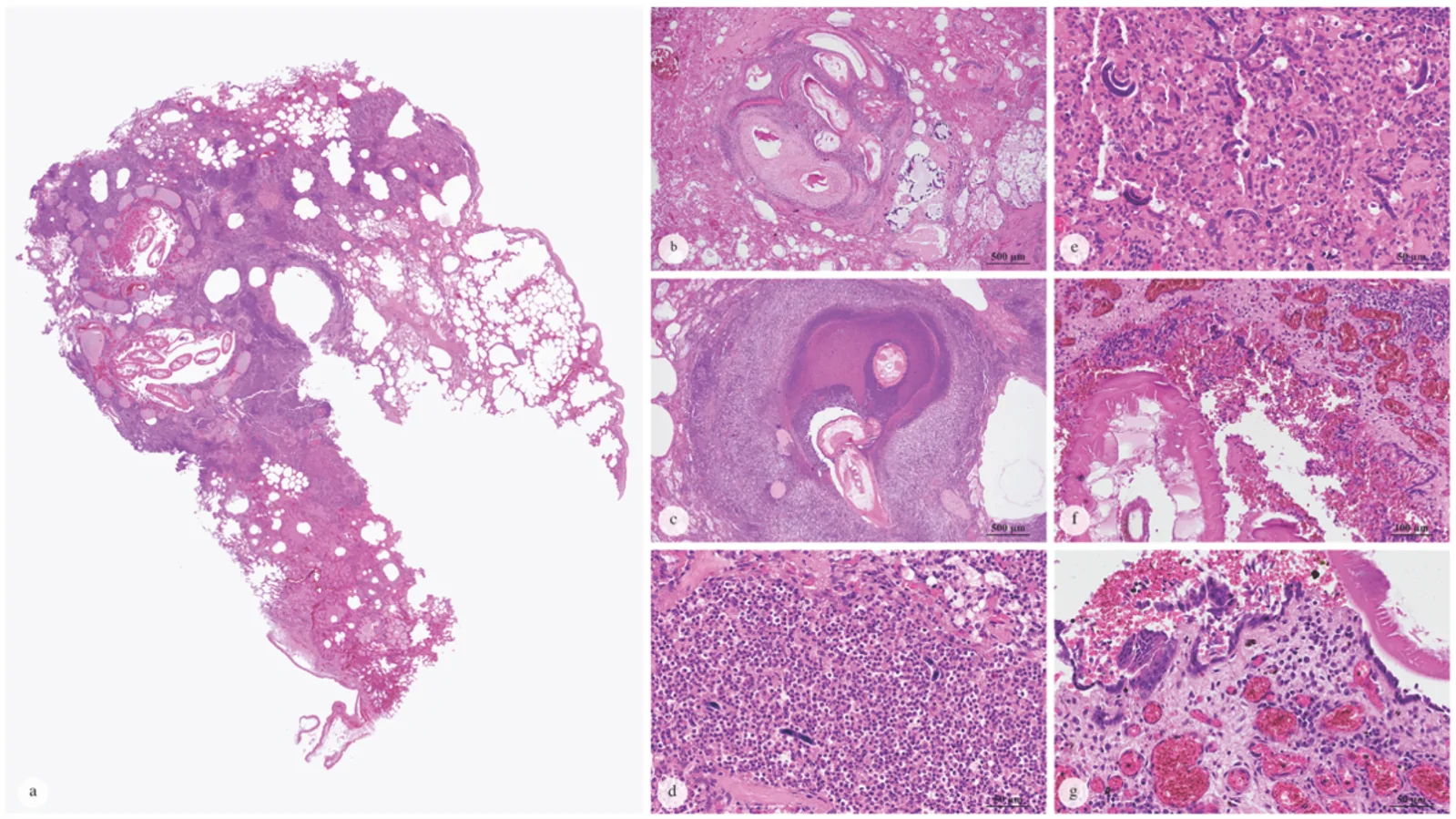
Histopathological alterations in the respiratory system associated with *Halocercus* spp. infection in stranded dolphins. (**a**) Low-power photomicrograph of the lung showing numerous adult nematodes within bronchi and bronchioles associated with severe verminous bronchointerstitial pneumonia. (**b**) Adult *Halocercus* spp. within a bronchiole surrounded by chronic inflammatory cell infiltration and peribronchiolar fibrosis. Dystrophic mineralization was present adjacent to the parasite, accompanied by alveolar collapse and inflammatory cell infiltration. (**c**) Pyogranuloma centered on a degenerating nematode within the pulmonary parenchyma. (**d**,**e**) Higher magnification of the pyogranulomatous lesion demonstrating numerous neutrophils and macrophages admixed with embedded larval nematodes. (**f**) Adult nematode within a bronchiole associated with severe bronchiolitis characterized by hemorrhage, congestion, inflammatory cell infiltration, and detachment of the respiratory epithelium. (**g**) Higher magnification of the bronchiolar lesion demonstrating epithelial detachment, congestion, hemorrhage, and inflammatory cell infiltration adjacent to the parasite. Hematoxylin and eosin stain. Scale bars as indicated. ((**a**–**g**) = H&E staining; original magnification: (**a**) = Subgross magnification, (**b**,**c**) = ×4, scale bar = 500 µm; (**f**) = ×20, scale bar = 100 µm; (**d**,**e**,**g**) = ×40, scale bar = 50 µm).

The respiratory tract exhibited the most prominent histopathological alterations among all examined organs (Figure 8). In TRCE076, numerous adult *Halocercus* spp. occupied the lumina of bronchi and bronchioles and were associated with severe chronic inflammatory responses characterized by dense infiltration of macrophages, lymphocytes, eosinophils, and fewer neutrophils surrounding affected airways (Figure 8a,b). Peribronchiolar fibrosis, pulmonary congestion, hemorrhage, collapse of adjacent alveoli, and occasional dystrophic mineralization adjacent to degenerating parasites were frequently observed.

Multiple pyogranulomas centered on degenerating adult nematodes and larval stages were present throughout the pulmonary parenchyma (Figure 8c–e). These lesions consisted predominantly of macrophages, neutrophils, eosinophils, multinucleated giant cells, and lymphocytes surrounding parasitic structures and necrotic cellular debris. Bronchiolar lesions were additionally characterized by epithelial detachment, hemorrhage, vascular congestion, and inflammatory cell infiltration adjacent to adult nematodes (Figure 8f,g). Collectively, these findings were consistent with severe chronic multifocal to locally extensive pyogranulomatous bronchopneumonia associated with *Halocercus* spp.

Similarly, pulmonary lesions in TRCE078 were comparatively less extensive and consisted primarily of multifocal bronchiolar inflammation associated with adult *Halocercus* spp., accompanied by pulmonary congestion and mild to moderate inflammatory cell infiltration. Histopathological evaluation of TRCE078 was additionally limited by mild postmortem autolysis, although parasite-associated pulmonary lesions remained readily identifiable.

#### 3.3.2. Gastrointestinal and Hepatobiliary System

**Figure 9:**
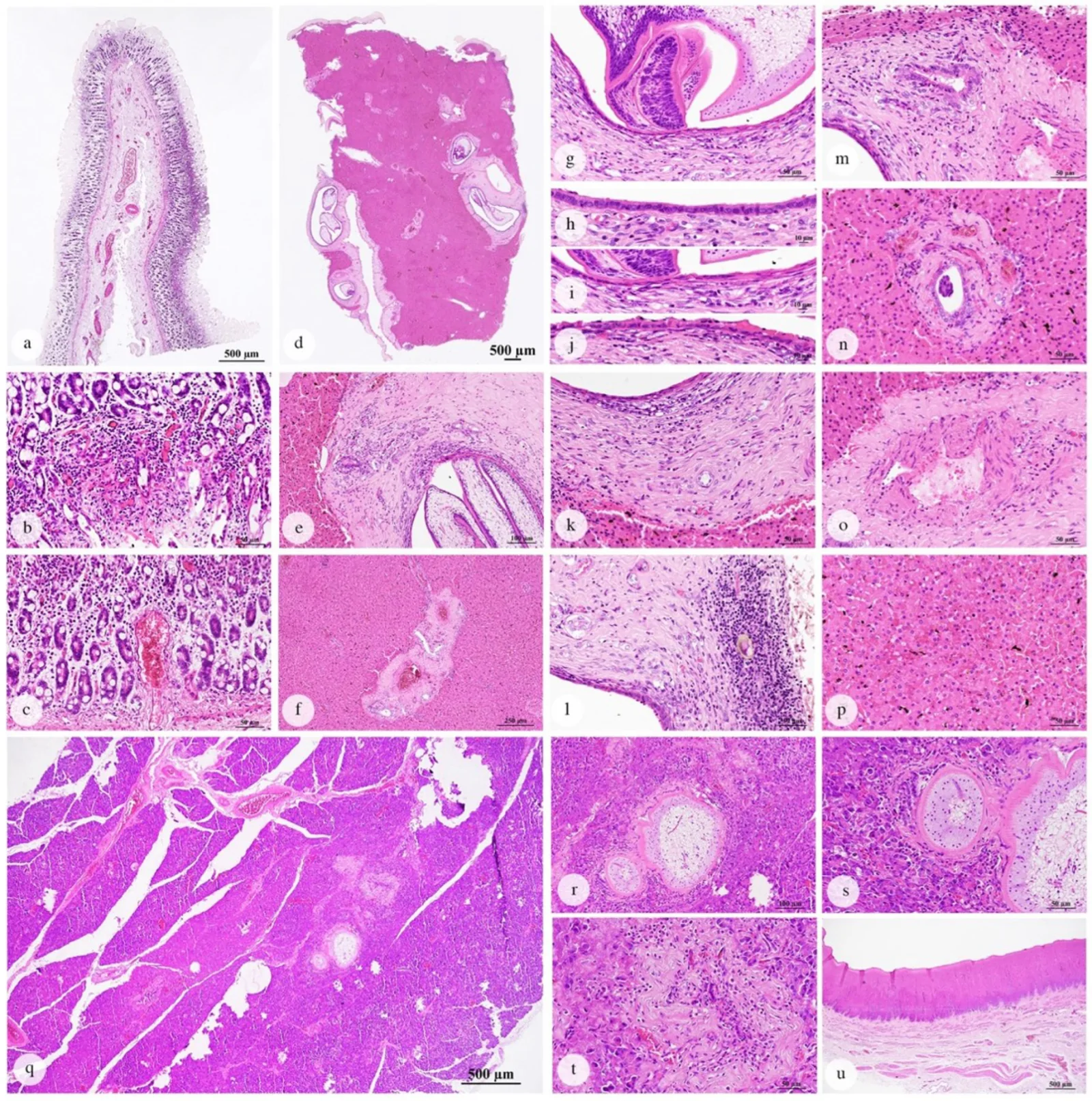
Histopathological alterations in the gastrointestinal, hepatobiliary, and pancreatic systems associated with multispecies helminth infections in stranded dolphins. (**a**) Low-power photomicrograph of the intestine showing chronic enteritis characterized by diffuse inflammatory cell infiltration within the mucosa. (**b**) Higher magnification of the intestinal mucosa demonstrating infiltration of lymphocytes and eosinophils accompanied by vascular congestion. (**c**) Marked vascular congestion within the intestinal wall. (**d**) Low-power photomicrograph of the liver showing a cestode consistent with *Clistobothrium* sp. within a bile duct. (**e**) Higher magnification demonstrating marked ductular reaction characterized by bile duct proliferation, periductal fibrosis, and chronic inflammatory cell infiltration surrounding an affected bile duct containing a parasite. (**f**) Secondary bile duct exhibiting epithelial hyperplasia, chronic inflammation, and vascular congestion. (**g**) Higher magnification showing attachment of the parasite sucker to the biliary epithelium, resulting in focal epithelial flattening consistent with mechanical injury. (**h**–**j**) Sequential biliary epithelial alterations adjacent to the parasite, including normal biliary epithelium (**h**), mechanical epithelial flattening (**i**), and epithelial degeneration characterized by blebbing and necrosis (**j**). (**k**) Mixed inflammatory infiltrate composed predominantly of eosinophils and lymphocytes within the ductal plate. (**l**) Parasite eggs embedded within the ductal plate associated with chronic inflammatory cell infiltration. (**m**) Eosinophilic cholangitis characterized by infiltration of eosinophils within the biliary epithelium. (**n**) Chronic portal cholangitis with inflammatory cell infiltration and epithelial casts within the lumen of a secondary bile duct. (**o**) Periarteriolar inflammation involving a portal artery. (**p**) Diffuse hepatic congestion without significant inflammatory change. (**q**) Pancreatic duct containing a helminth associated with periductal inflammation, focal pancreatic necrosis, and fibrosis. (**r**) Higher magnification of the parasite within the pancreatic duct demonstrating adjacent tissue injury. (**s**) Eosinophil-rich inflammatory infiltrate surrounding the affected pancreatic duct. (**t**) Chronic eosinophilic inflammation with associated periductal fibrosis. (**u**) Stomach without significant histopathological alterations. Hematoxylin and eosin stain. Scale bars as indicated. ((**a**–**u**) = H&E staining; original magnification: (**a**,**d**) = Subgross magnification, (**q**) = ×4, scale bar = 500 µm; (**f**,**r**) = ×10, scale bar = 250 µm; (**e**) = ×20, scale bar = 100 µm; (**b**,**c**,**g**,**k**–**p**,**s**–**u**) = ×40, scale bar = 50 µm; crop pictures: (**h**–**j**) = ×40, scale bar = 10 µm).

Histopathological alterations within the gastrointestinal and hepatobiliary systems were less extensive than those observed in the respiratory tract but demonstrated distinct parasite-associated inflammatory responses (Figure 9). In TRCE076, adult *Tetrabothrius* spp. within the intestinal lumen were associated with moderate chronic enteritis characterized by diffuse vascular congestion and multifocal infiltration of lymphocytes, eosinophils, and fewer macrophages within the mucosa, extending into the submucosa (Figure 9a–c).

The most prominent hepatobiliary lesions were associated with cestodes consistent with *Clistobothrium* sp. within the lumina of intrahepatic and secondary bile ducts (Figure 9d–g). Affected bile ducts exhibited marked ductular reaction characterized by bile duct proliferation, periductal fibrosis, and chronic inflammatory cell infiltration composed predominantly of lymphocytes and eosinophils (Figure 9e,f). Direct contact and attachment of the cestodes to the biliary epithelium were associated with progressive epithelial injury, ranging from focal epithelial flattening to cytoplasmic blebbing and epithelial necrosis (Figure 9g–j). Parasite eggs embedded within the ductal plate elicited focal inflammatory reactions, while eosinophilic cholangitis, epithelial casts within secondary bile ducts, and periarteriolar inflammation were observed within affected portal tracts (Figure 9k–o). Hepatic congestion was also evident but was not consistently accompanied by significant inflammation in the adjacent hepatic parenchyma (Figure 9p). In TRCE078, cestodes consistent with *Clistobothrium* sp. were additionally identified within pancreatic ducts and were associated with chronic eosinophilic periductal inflammation, fibrosis, and focal necrosis of the surrounding pancreatic parenchyma (Figure 9q–t). Histological examination of the stomach revealed no significant parasite-associated inflammation or tissue destruction (Figure 9u).

#### 3.3.3. Subcutaneous and Reproductive Systems

**Figure 10:**
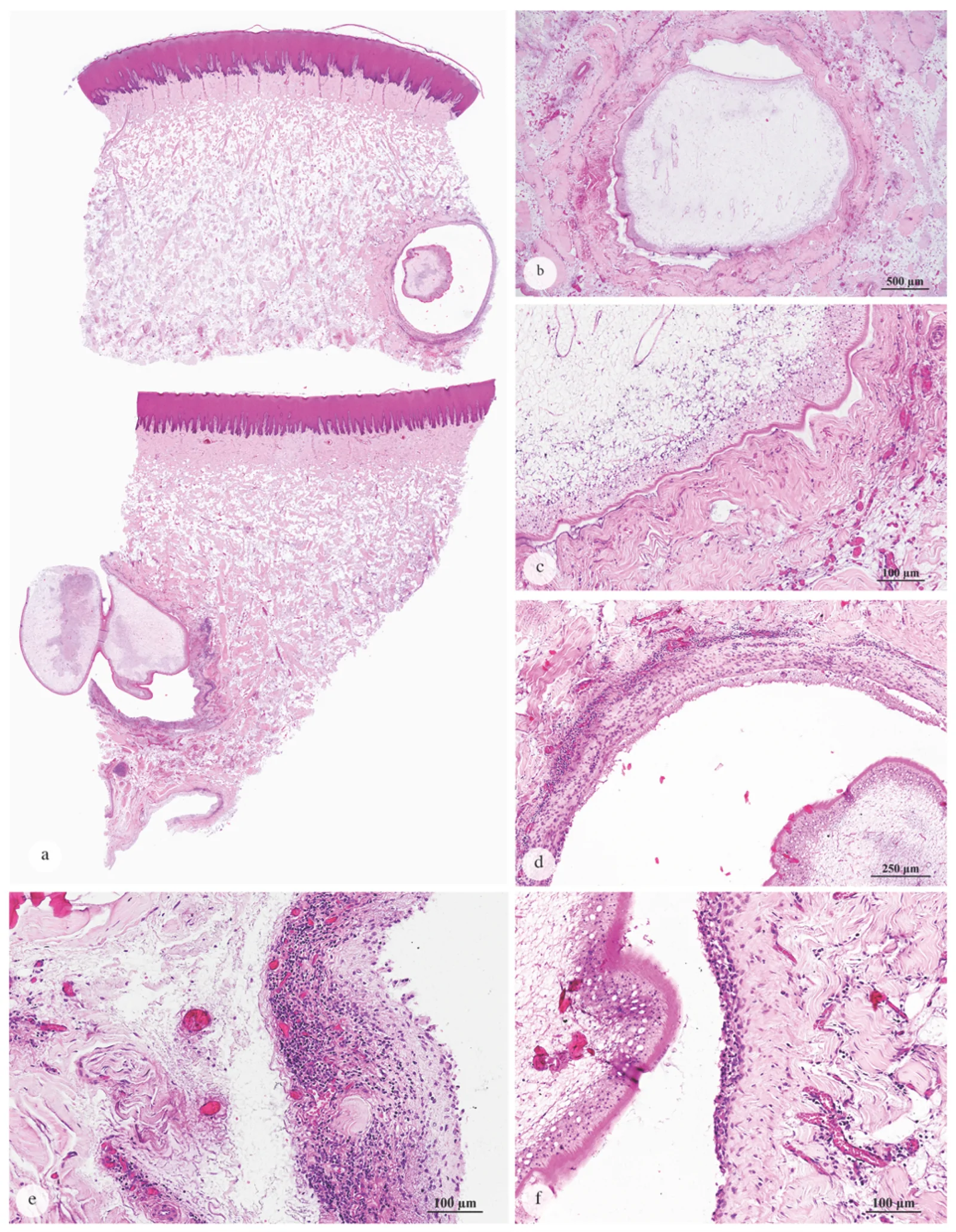
Histopathological alterations in the blubber associated with larval cestode infection in stranded dolphins. (**a**) Low-power photomicrograph of the blubber showing two parasitic cysts embedded within the subcutaneous adipose tissue. (**b**) Higher magnification demonstrating a larval cestode occupying the cystic cavity and surrounded by a chronic inflammatory reaction. (**c**) Higher magnification of the outer cyst wall showing a dense fibrous connective tissue capsule with prominent neovascularization. (**d**) Cross-section of the parasitic cyst demonstrating an incomplete fibrous capsule. The subcapsular region exhibited mild to moderate infiltration of mononuclear inflammatory cells with fewer neutrophils, whereas the cystic cavity contained the parasite admixed with abundant proteinaceous material. (**e**) Higher magnification of the cyst capsule showing infiltration of mononuclear inflammatory cells and early fibroplasia. (**f**) Perivascular inflammation (perivasculitis) characterized by inflammatory cell infiltration surrounding small blood vessels adjacent to the parasitic cyst. Hematoxylin and eosin stain. Scale bars as indicated. ((**a**–**f**) = H&E staining; original magnification: (**a**) = Subgross magnification, (**b**) = ×4, scale bar = 500 µm; (**d**) = ×10, scale bar = 250 µm; (**c**,**e**,**f**) = ×20, scale bar = 100 µm).

Histopathological lesions within the subcutaneous tissue were characterized by mild chronic multifocal parasitic cysts associated with localized inflammatory reactions (Figure 10). Larval cestode cysts were partially enclosed by thin to moderately developed fibrous connective tissue capsules. The subcapsular region exhibited mild to moderate infiltration of mononuclear inflammatory cells admixed with fewer neutrophils, whereas the cystic cavity contained the parasite embedded within abundant eosinophilic proteinaceous material (Figure 10a–e). Mild perivasculitis characterized by perivascular infiltration of mononuclear inflammatory cells was frequently observed in blood vessels adjacent to parasitic cysts (Figure 10f). Apart from the localized lesions surrounding the cysts and adjacent vessels, the surrounding subcutaneous adipose tissue was largely unremarkable.

Similar parasite-associated lesions were identified within the testicular interstitium, where larval cestode cysts were surrounded by partial fibrous encapsulation accompanied by mild multifocal mononuclear inflammatory cell infiltration. The adjacent seminiferous tubules remained largely preserved without significant degenerative change.

#### 3.3.4. Other Organs

Histological examination of the heart, kidney, spleen, and lymph nodes revealed predominantly vascular congestion and varying degrees of postmortem autolysis, whereas the remaining tissues showed no significant parasite-associated inflammatory lesions or tissue destruction.

#### 3.3.5. Pathological Assessment of Mortality

Based on the integrated gross and histopathological findings, severe parasite-associated pulmonary disease was considered a potentially important contributor to the acute clinical deterioration and death, particularly in TRCE076. The extensive burden of *Halocercus* spp. within the tracheobronchial tree, together with abundant airway exudate, partial luminal obstruction, widespread pulmonary consolidation, and severe chronic pyogranulomatous bronchopneumonia, could have substantially compromised respiratory function. In TRCE078, although numerous pulmonary nematodes and marked gross pulmonary consolidation were also present, the associated histopathological lesions were comparatively less extensive, making their contribution to mortality less certain. Other parasite-associated lesions in the gastrointestinal, hepatobiliary, pancreatic, blubber, and reproductive tissues were predominantly chronic and localized and were therefore considered less likely to have directly accounted for the acute deterioration. No severe traumatic lesions or other major pathological processes sufficient to independently explain death were identified. Nevertheless, because of the limited ante-mortem diagnostic data and the multifactorial nature of cetacean strandings, a definitive cause of death could not be established in either dolphin.

### 3.4. Immunohistochemical Findings in Helminth-Affected Tissues

**Figure 11:**
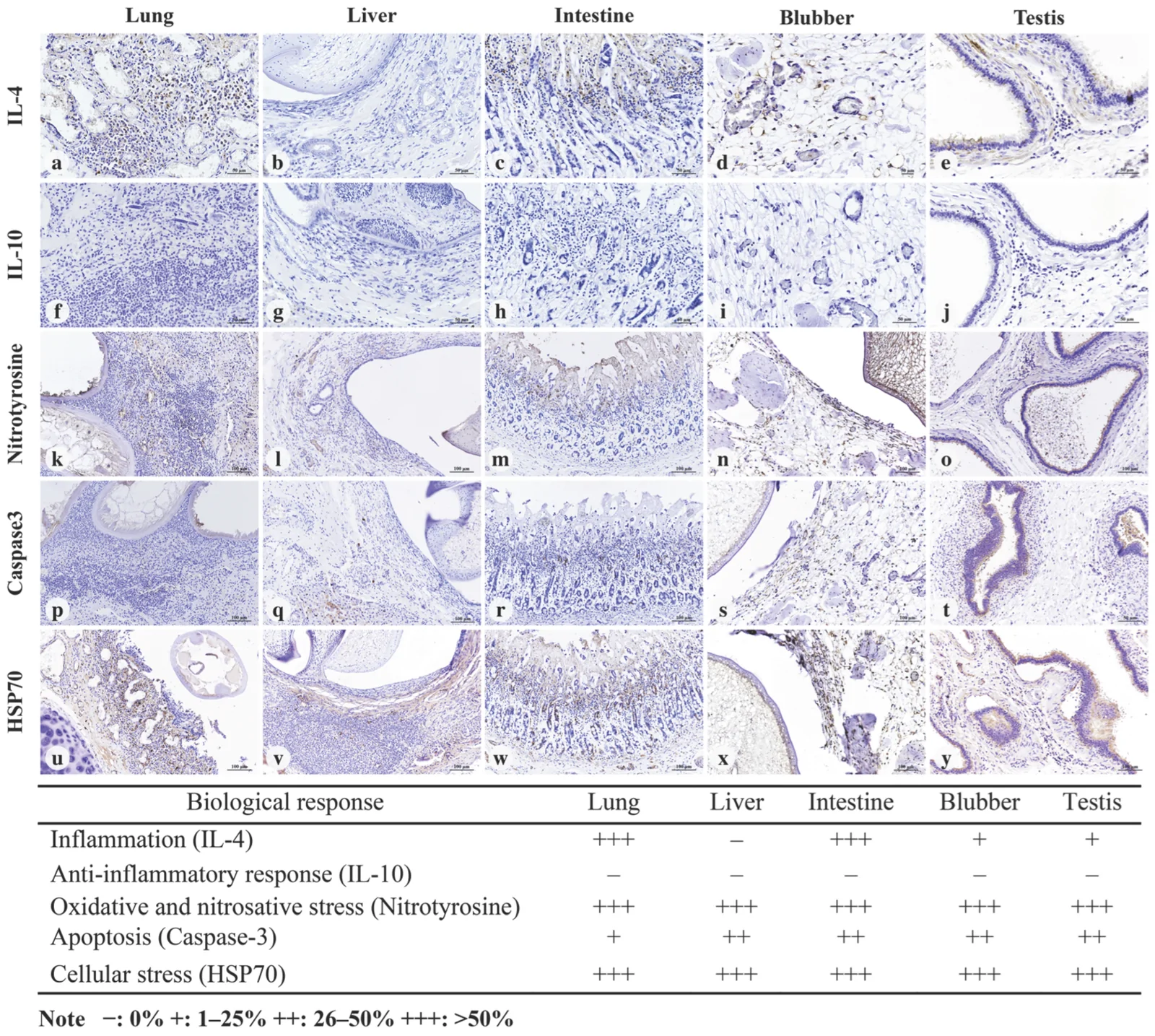
Immunohistochemical evidence of inflammatory, oxidative stress, apoptotic, and cellular stress responses associated with multispecies helminth infections in stranded dolphins. Representative immunohistochemical staining of IL-4, IL-10, nitrotyrosine, caspase-3, and HSP70 in the lung, liver, intestine, blubber, and testis. (**a**–**e**) IL-4 immunostaining. Extensive immunoreactivity (+++) was observed in inflammatory cells within the pulmonary interstitium and surrounding bronchi and bronchioles (**a**) and within the intestinal mucosa and lamina propria (**c**). Immunoreactivity was also detected in inflammatory cells surrounding blood vessels within the blubber (**d**) and interstitial inflammatory cells adjacent to seminiferous tubules in the testis (**e**). No detectable IL-4 immunoreactivity was observed in the liver (**b**). (**f**–**j**) IL-10 immunostaining. No detectable immunoreactivity (−) was observed in any examined tissue. (**k**–**o**) Nitrotyrosine immunostaining. Immunoreactivity was observed in inflammatory cells, bronchial epithelium, and nematode tegument within the lung (**k**); bile duct epithelium and portal vascular structures in the liver (**l**); intestinal mucosa and lamina propria (**m**); inflammatory cells and larval cestode tegument in the blubber (**n**); and seminiferous epithelium in the testis (**o**). (**p**–**t**) Caspase-3 immunostaining. Comparatively limited immunoreactivity was observed in the lung (**p**), whereas positive labeling was detected in bile duct epithelium and adjacent hepatocytes (**q**), intestinal mucosal epithelial cells (**r**), endothelial cells and adipocytes within the blubber (**s**), and seminiferous epithelial cells (**t**). (**u**–**y**) HSP70 immunostaining. Extensive immunoreactivity was observed in inflammatory, stromal, interstitial, and epithelial cells throughout the examined organs. Brown DAB labeling indicates positive immunoreactivity. Hematoxylin counterstain. Scale bars = 100 μm unless otherwise indicated.

Immunohistochemical analyses demonstrated distinct inflammatory, oxidative/nitrosative stress, apoptotic, and cellular stress responses in tissues affected by multispecies helminth infections (Figure 11). The extent and cellular distribution of immunoreactivity varied among organs and generally corresponded with the distribution of parasite-associated lesions identified histologically.

IL-4 immunoreactivity was most extensive in the respiratory and intestinal tissues. In the lung, IL-4 immunoreactivity was observed in >50% of evaluated cells (+++), predominantly involving inflammatory cells within the pulmonary interstitium and surrounding bronchi and bronchioles affected by *Halocercus* infection (Figure 11a). Similarly, IL-4 immunoreactivity involving >50% of evaluated cells (+++) was detected within the intestinal mucosa and inflammatory cells of the lamina propria associated with adult *Tetrabothrius* infection (Figure 11c). In the blubber and testis, IL-4 immunoreactivity involved a smaller proportion of cells, predominantly inflammatory cells surrounding blood vessels within the blubber and interstitial inflammatory cells adjacent to seminiferous tubules in the testis (Figure 11d,e). In contrast, no detectable IL-4 immunoreactivity was observed in the liver (Figure 11b). IL-10 immunoreactivity was absent (−) in all examined tissues (Figure 11f–j).

Nitrotyrosine immunoreactivity was widely distributed among tissues harboring helminths (Figure 11k–o). In the lung, extensive immunoreactivity was observed in inflammatory cells, bronchial epithelium, and the tegument of *Halocercus* spp. (Figure 11k). Positive labeling was also localized to bile duct epithelium and vascular structures within portal areas of the liver (Figure 11l), intestinal mucosa and lamina propria (Figure 11m), inflammatory cells and the tegument of larval cestodes within the blubber (Figure 11n), and seminiferous epithelium of the testis (Figure 11o). These findings demonstrated widespread protein nitration consistent with oxidative/nitrosative stress across parasite-associated tissues.

Caspase-3 immunoreactivity was detected in all examined organs, although the proportion of immunoreactive cells varied among tissues (Figure 11p–t). The lung exhibited comparatively limited caspase-3 immunoreactivity despite the presence of extensive inflammatory lesions (Figure 11p). In contrast, greater proportions of immunoreactive cells were observed in bile duct epithelium and adjacent hepatocytes in the liver (Figure 11q), epithelial cells of the intestinal mucosa (Figure 11r), endothelial cells and adipocytes within the blubber (Figure 11s), and seminiferous epithelial cells of the testis (Figure 11t).

HSP70 immunoreactivity was widely distributed throughout the examined parasite-associated tissues (Figure 11u–y). Extensive cytoplasmic labeling was observed in inflammatory cells, stromal connective tissue, interstitial cells, and epithelial cells across the examined organs, consistent with a widespread cellular stress response associated with parasite-affected tissues. This analysis demonstrated prominent IL-4 immunoreactivity with no detectable IL-10 immunoreactivity, widespread nitrotyrosine and HSP70 labeling, and tissue-dependent caspase-3 immunoreactivity across organs affected by multispecies helminth infections.

## 4. Discussion

Helminth infections are commonly encountered in stranded cetaceans and may occur either as single infections or concurrently with other parasite taxa [30,47,48]. In the present study, both stranded dolphins harbored multispecies helminth infections involving multiple anatomical sites. The spinner dolphin (*S. longirostris*) exhibited greater parasite diversity and anatomical distribution, with *H. delphini* involving the respiratory tract, *Tetrabothrius* sp. in the intestine, and *Clistobothrium* sp. in the liver, blubber, and testis. The pantropical spotted dolphin (*S. attenuata*) harbored *H. delphini* in the respiratory tract and *Clistobothrium* sp. in the blubber and testis. Concurrent helminth infections have previously been documented in several stranded cetacean species. A neonatal Risso’s dolphin (*Grampus griseus*) from the western Mediterranean, for example, harbored both *H. delphini* and *Stenurus globicephalae* within the respiratory system [35], while a stranded Blainville’s beaked whale (*Mesoplodon densirostris*) examined in British waters was concurrently infected with *Anisakis simplex* and the intestinal cestode *Tetrabothrius* sp. [47]. Multiple helminth taxa, including *S. globicephalae* and the larval phyllobothriid cestodes *Clistobothrium delphini* and *C. grimaldii*, have also been reported in long-finned pilot whales (*Globicephala melas*) from the western Mediterranean [49]. More broadly, *Halocercus* spp. have been documented in the respiratory tract of several odontocetes, including common, striped, and bottlenose dolphins and killer whales [49,50,51], whereas larval *Clistobothrium* spp. have been reported from the subcutaneous blubber and other tissues of common and striped dolphins and long-finned pilot whales [49,51]. Thus, the parasite taxa and anatomical predilection sites observed in the present cases were broadly consistent with previous reports. However, to our knowledge, this study provides the first integrated characterization of heavy polyparasitism involving multiple helminth taxa across several organ systems within individual *Stenella* dolphins, supported by morphological, molecular, and pathological evidence.

Morphological identification was further supported by molecular and phylogenetic analyses. The pulmonary nematodes exhibited morphological features consistent with *Halocercus* spp., including characteristic male and female reproductive structures and gravid females containing intrauterine eggs and larvae. ITS2 sequencing subsequently identified isolates from both dolphins as *H. delphini*, showing 98.73–99.38% nucleotide identity with reference sequences and clustering within the *H. delphini* clade, distinct from other pseudaliid taxa. This phylogenetic placement is consistent with previous molecular and taxonomic studies supporting the synonymy of *Skrjabinalius guevarai* with *H. delphini* [52]. *H. delphini* has been reported from several delphinid hosts and geographical regions, including common dolphins (*Delphinus delphis*) and multiple *Stenella* spp. across Atlantic, Mediterranean, and Pacific waters [52]. More recent studies have further documented pulmonary *H. delphini* infection in common dolphins from northern Spain [53], striped dolphins (*Stenella coeruleoalba*) from the western Mediterranean [54], and molecularly confirmed *H. delphini* in striped dolphins from the western Mediterranean [49]. However, the limited number of specimens and available reference sequences in the present study preclude conclusions regarding host specificity or geographical population structure. Partial 18S rRNA sequencing identified five extraintestinal cestode specimens as *Clistobothrium* sp., with >99.5% nucleotide similarity among the examined sequences. *Clistobothrium* sp. was identified from the liver, blubber, and testis of the spinner dolphin and from the blubber and testis of the pantropical spotted dolphin. Comparable extraintestinal distributions have been reported in other odontocetes, including *C. delphini* and *C. grimaldii* in the subcutaneous blubber, peritoneum, and abdominal tissues of common and striped dolphins [51], and in the blubber of long-finned pilot whales (*Globicephala melas*) [49]. The intestinal cestode from the spinner dolphin was identified as *Tetrabothrius* sp. and clustered within the *Tetrabothrius* clade. Nevertheless, species-level identification of both cestodes remains uncertain because of the limited availability and discriminatory resolution of comparable 18S rRNA reference sequences in GenBank. Therefore, their conservative identification as *Clistobothrium* sp. and *Tetrabothrius* sp. is warranted, and additional molecular markers may provide greater species-level resolution.

Histopathological examination demonstrated that the severity and nature of parasite-associated lesions varied according to parasite location and the affected organ. The most substantial alterations occurred in the respiratory tract, where *H. delphini* infection was associated predominantly with severe chronic pyogranulomatous bronchopneumonia involving both adult and larval nematodes. In contrast, lesions associated with cestodes were generally more localized. Intestinal *Tetrabothrius* infection in *S. longirostris* was associated with chronic lymphocytic and eosinophilic enteritis, whereas cestodes consistent with *Clistobothrium* sp. within the hepatobiliary and pancreatic ductal systems were associated with epithelial injury, eosinophilic and lymphocytic inflammation, ductular reaction, fibrosis, and focal tissue necrosis. Larval *Clistobothrium* cysts in the blubber elicited comparatively limited reactions, characterized primarily by fibrous encapsulation and mild to moderate local inflammation. These pathological patterns are broadly consistent with previous observations of helminth infections in cetaceans and other mammalian hosts. Pulmonary infection with *H. delphini* and other *Halocercus* spp. has been associated with bronchitis, bronchopneumonia, granulomatous or bronchointerstitial pneumonia, pulmonary fibrosis, and alterations of the surrounding pulmonary parenchyma [50,51,53]. The severity of pulmonary lesions may vary considerably among infected cetaceans, potentially reflecting differences in parasite burden and intrapulmonary distribution [54], while severe infections may also be complicated by secondary bacterial pneumonia [51]. Gastrointestinal cestodes, including *Tetrabothrius* spp., are recognized parasites of cetaceans and may be associated with localized intestinal lesions [55]. Comparable inflammatory and fibrotic responses to duct-associated cestodes have also been described in other mammalian hosts, including cholangitis and biliary fibrosis associated with *Stilesia hepatica* in ruminants [56] and eosinophilic to pyogranulomatous hepatic inflammation associated with *Mesocestoides* infection in dogs [57]. In contrast, larval phyllobothriid cestodes encysted within cetacean blubber and other extraintestinal tissues may elicit relatively limited host reactions [47], consistent with the localized changes observed around *Clistobothrium* cysts in the present cases. Collectively, these findings indicate that polyparasitism was associated with a spectrum of pathological alterations ranging from localized chronic tissue reactions to severe pulmonary disease, with the nature and severity of lesions differing according to parasite location, developmental stage, and interaction with surrounding tissues. The severe pulmonary lesions, particularly in TRCE076, may have compromised respiratory function and contributed to the acute deterioration and death; however, a definitive cause of death could not be established.

To further characterize the biological responses associated with polyparasitism, immunohistochemistry was used to assess inflammatory, oxidative/nitrosative stress, cellular stress, and apoptotic responses in parasite-affected tissues. Overall, IL-4 immunoreactivity was most extensive in the lung and intestine, corresponding to prominent chronic and eosinophilic inflammation associated with *Halocercus* and *Tetrabothrius* infections, whereas IL-10 was not detected in the examined tissues. IL-4 is a key cytokine associated with type 2 immunity against helminths and is closely linked to eosinophilic inflammation and tissue responses to multicellular parasites [58]. Thus, the IL-4 pattern observed in the present cases is compatible with a localized type 2-associated response. In contrast, although IL-10 has an important immunoregulatory role during chronic helminth infection [59], its absence should be interpreted cautiously because tissue expression may depend on infection stage, local cellular composition, and assay sensitivity. Nitrotyrosine and HSP70 immunoreactivity were widely distributed across parasite-associated tissues, supporting the occurrence of oxidative/nitrosative and cellular stress, respectively. Nitrotyrosine reflects protein nitration associated with reactive oxygen and nitrogen species, which may be generated during persistent parasite-associated inflammation and contribute to local tissue injury [60,61], whereas HSP70 is involved in cellular adaptation to stress and maintenance of protein homeostasis under adverse conditions [62,63]. Caspase-3 was also detected across the examined organs but showed a tissue-dependent distribution, indicating that apoptotic responses varied among affected tissues; however, caspase-3 immunoreactivity alone cannot identify the initiating stimulus of apoptosis [64]. Collectively, these findings suggest that multispecies helminth infections were associated with overlapping biological responses involving type 2-associated inflammation, oxidative/nitrosative stress, cellular stress, and apoptosis. Rather than establishing a sequential causal pathway, the combined histopathological and immunohistochemical findings support a pathological framework in which persistent parasite–tissue interactions are accompanied by local inflammation and tissue injury together with oxidative/nitrosative and cellular stress, while apoptotic responses vary according to the affected tissue. An important limitation of this study was the absence of non-infected tissues from healthy cetaceans for use as biological baseline controls. Although the spatial localization of immunoreactivity within and around parasite-associated lesions supports an association between parasitic infection and the observed cellular responses, it cannot establish that these responses were exclusively parasite-induced. In particular, HSP70 and caspase-3 expression may also have been influenced by terminal stranding-related stress or other systemic processes. Therefore, these immunohistochemical findings should be interpreted as lesion-associated cellular responses rather than definitive evidence of parasite-specific effects.

## 5. Conclusions

This study highlights polyparasitism as a multisystem pathological condition in stranded dolphins rather than merely the concurrent presence of multiple helminth taxa. Integrating parasitological, molecular, pathological, and immunohistochemical evidence demonstrated that the pathological significance of helminth infection is closely linked to parasite distribution, tissue interaction, and the accompanying host response. This integrated perspective provides a useful framework for interpreting helminth infections during cetacean postmortem investigations and emphasizes the importance of evaluating parasite burden in the context of associated tissue alterations. Although the contribution of polyparasitism to stranding or mortality cannot be determined from these cases alone, its potential impact on the health of affected dolphins warrants greater consideration in marine mammal disease surveillance and pathological assessment.

## Figures and Tables

**Table 1:** Basic characteristics, body condition, and stranding history of the two live-stranded dolphins examined in this study.

Characteristic	TRCE076	TRCE078
Species	Spinner dolphin (*Stenella longirostris*)	Pantropical spotted dolphin (*Stenella attenuata*)
Sex	Male	Male
Developmental stage	Subadult	Adult
Total body length (cm)	178	200
Body weight (kg)	43.8	70.0
Body condition score	2.5/5	3/5
Body condition	Relatively thin	Moderate
Stranding status	Live-stranded	Live-stranded
Postmortem condition	Fresh	Fresh

**Table 2:** Distribution of helminth species identified in two stranded dolphins. Both dolphins harbored concurrent infections with multiple helminth taxa. The spinner dolphin (*Stenella longirostris*, TRCE076) exhibited a broader anatomical distribution of parasites involving the respiratory, gastrointestinal, hepatobiliary, reproductive, and subcutaneous tissues, whereas the pantropical spotted dolphin (*Stenella attenuata*, TRCE078) showed infections primarily affecting the respiratory tract, blubber, and testis.

Helminth Taxon	TRCE076 (*S. longirostris*)	TRCE078 (*S. attenuata*)	Predominant Anatomical Location
*Halocercus delphini*	✓	✓	Trachea and lung
*Tetrabothrius* sp.	✓	–	Intestine
*Clistobothrium* sp.	✓	✓	Blubber
*Clistobothrium* sp.	✓	–	Liver
*Clistobothrium* sp.	✓	✓	Testis

Note: ✓, present; –, not detected.

## Data Availability

The data presented in this study are available on request from the corresponding author (W.S.).

## Abbreviations

The following abbreviations are used in this manuscript:

18S rRNA	18S ribosomal ribonucleic acid
BSA	Bovine serum albumin
DAB	3,3′-Diaminobenzidine
DNA	Deoxyribonucleic acid
H&E	Hematoxylin and eosin
HSP70	Heat shock protein 70
IHC	Immunohistochemistry
IL-4	Interleukin-4
IL-10	Interleukin-10
ITS2	Internal transcribed spacer 2
MAFFT	Multiple Alignment using Fast Fourier Transform
MEGA	Molecular Evolutionary Genetics Analysis
NCBI	National Center for Biotechnology Information
PBS	Phosphate-buffered saline
PCR	Polymerase chain reaction
TBS	Tris-buffered saline

## References

[1] Kiszka J.J., Woodstock M.S., Heithaus M.R. (2022). Functional roles and ecological importance of small cetaceans in aquatic ecosystems. Front. Mar. Sci..

[2] Yang S., Li S., Jin Y., Liu Z. (2023). Changing trends in cetacean strandings in the East China Sea: Identifying relevant variables and implications for conservation and management. Diversity.

[3] Kirschvink J.L., Dizon A.E., Westphal J.A. (1986). Evidence from strandings for geomagnetic sensitivity in cetaceans. J. Exp. Biol..

[4] Brabyn M.W., McLean I.G. (1992). Oceanography and coastal topography of herd-stranding sites for whales in New Zealand. J. Mammal..

[5] Evans K., Thresher R., Warneke R.M., Bradshaw C.J.A., Pook M., Thiele D., Hindell M.A. (2005). Periodic variability in cetacean strandings: Links to large-scale climate events. Biol. Lett..

[6] Zellar R., Pulkkinen A., Moore K., Rousseaux C.S., Reeb D. (2021). Oceanic and atmospheric correlations to cetacean mass stranding events in Cape Cod, Massachusetts, USA. Geophys. Res. Lett..

[7] Weilgart L.S. (2007). The impacts of anthropogenic ocean noise on cetaceans and implications for management. Can. J. Zool..

[8] Guan S., Brookens T. (2023). An overview of research efforts to understand the effects of underwater sound on cetaceans. Water Biol. Secur..

[9] Schaap I., Buedenbender L., Johann S., Hollert H., Dogruer G. (2023). Impact of chemical pollution on threatened marine mammals: A systematic review. J. Hazard. Mater..

[10] Bearzi G., Eddy L., Piwetz S., Reggente M., Cozzi B., Vonk J., Shackelford T. (2017). Cetacean behavior toward the dead and dying. Encyclopedia of Animal Cognition and Behavior.

[11] Díaz-Delgado J., Fernández A., Sierra E., Sacchini S., Andrada M., Vela A.I., Quesada-Canales Ó., Paz Y., Zucca D., Groch K. (2018). Pathologic findings and causes of death of stranded cetaceans in the Canary Islands (2006–2012). PLoS ONE.

[12] Garcia-Bustos V., Acosta-Hernández B., Cabañero-Navalón M.D., Ruiz-Gaitán A.C., Pemán J., Rosario Medina I. (2024). Potential fungal zoonotic pathogens in cetaceans: An emerging concern. Microorganisms.

[13] Bordes F., Morand S. (2011). The impact of multiple infections on wild animal hosts: A review. Infect. Ecol. Epidemiol..

[14] Al-Amin A.S.M., Wadhwa R. (2023). Helminthiasis. StatPearls.

[15] Oliveira J.B., Morales J.A., González-Barrientos R.C., Hernández-Gamboa J., Hernández-Mora G. (2011). Parasites of cetaceans stranded on the Pacific coast of Costa Rica. Vet. Parasitol..

[16] Vongpakorn M., Pongsopawijit P., Manawatthana S., Soison O., Adulyanukosol K., Kittiwatanawong K., Boontong P. Identification of helminths in dolphins stranded along the Andaman coast of Thailand during 1992–2006. Proceedings of the Thailand-Japan Joint Conference on Animal Health 2012: The 25th Year Anniversary of National Institute of Animal Health.

[17] Aguilar-Aguilar R., Moreno R., Salgado-Maldonado G., Villa-Ramírez B. (2001). Gastrointestinal helminths of spinner dolphins *Stenella longirostris* (Gray, 1828) (Cetacea: Delphinidae) stranded in La Paz Bay, Baja California Sur, Mexico. Comp. Parasitol..

[18] Dailey M., Perrin W. (1973). Helminth parasites of porpoises of the genus *Stenella* in the eastern tropical Pacific, with descriptions of two new species: *Mastigonema stenellae* gen. et sp. n. (Nematoda: Spiruroidea) and *Zalophotrema pacificum* sp. n. (Trematoda: Digenea). Fish. Bull..

[19] Fernández M., Balbuena J.A., Raga J.A. (1994). *Hadwenius tursionis* (Marchi, 1873) n. comb. (Digenea, Campulidae) from the bottlenose dolphin *Tursiops truncatus* (Montagu, 1821) in the western Mediterranean. Syst. Parasitol..

[20] Jaber J.R., Pérez J., Arbelo M., Zafra R., Fernández A. (2006). Pathological and immunohistochemical study of gastrointestinal lesions in dolphins stranded in the Canary Islands. Vet. Rec..

[21] Dailey M.D., Dierauf L.A., Gulland F.M.D. (2001). Parasitic diseases. CRC Handbook of Marine Mammal Medicine.

[22] Moreau E., Chauvin A. (2010). Immunity against helminths: Interactions with the host and the intercurrent infections. BioMed Res. Int..

[23] Wakelin D., Baron S. (1996). Helminths: Pathogenesis and defenses. Medical Microbiology.

[24] Wynn T.A., Allen J.E., Kaufmann S.H.E., Rouse B.T., Sacks D.L. (2011). Pathogenesis of helminth infections. The Immune Response to Infection.

[25] Suyapoh W., Tirnitz-Parker J.E.E., Tangkawattana S., Suttiprapa S., Sripa B. (2021). Biliary migration, colonization, and pathogenesis of *O. viverrini* co-infected with CagA+ *Helicobacter pylori*. Pathogens.

[26] Smout M.J., Sripa B., Laha T., Mulvenna J., Gasser R.B., Young N.D., Bethony J.M., Brindley P.J., Loukas A. (2011). Infection with the carcinogenic human liver fluke, *Opisthorchis viverrini*. Mol. BioSyst..

[27] Suyapoh W., Tangkawattana S., Suttiprapa S., Punyapornwithaya V., Tangkawattana P., Sripa B. (2021). Synergistic effects of cagA+ *Helicobacter pylori* co-infected with *Opisthorchis viverrini* on hepatobiliary pathology in hamsters. Acta Trop..

[28] Abd Ellah M.R. (2013). Involvement of free radicals in parasitic infestations. J. Appl. Anim. Res..

[29] Jaber J.R., Zafra R., Pérez J., Suárez-Bonnet A., González J.F., Carrascosa C., Andrada M., Arbelo M., Fernández A. (2013). Immunopathological study of parasitic cholangitis in cetaceans. Res. Vet. Sci..

[30] Suárez-González Z., González J.F., Arbelo M., Sierra E., Castro-Alonso A., Hernández J.N., Martín V., Fraija-Fernández N., Fernández A. (2024). Parasitic infections in stranded whales and dolphins in Canary Islands (2018–2022): An update. Animals.

[31] Dailey M., Stroud R. (1978). Parasites and associated pathology observed in cetaceans stranded along the Oregon coast. J. Wildl. Dis..

[32] Pugliares-Bonner K., Bogomolni A., Touhey K., Herzig S., Harry C., Moore M. (2007). Marine Mammal Necropsy: An Introductory Guide for Stranding Responders and Field Biologists.

[33] Muller R., Wakelin D. (2002). Worms and Human Disease.

[34] Mejías-Alpízar M.J., Porras-Silesky C., Rodríguez E.J., Quesada J., Alfaro-Segura M.P., Robleto-Quesada J., Gutiérrez R., Rojas A. (2024). Mitochondrial and ribosomal markers in the identification of nematodes of clinical and veterinary importance. Parasites Vectors.

[35] Pool R., Romero-Rubira C., Raga J.A., Fernández M., Aznar F.J. (2021). Determinants of lungworm specificity in five cetacean species in the western Mediterranean. Parasites Vectors.

[36] Škeříková A., Hypša V., Scholz T. (2001). Phylogenetic analysis of European species of *Proteocephalus* (Cestoda: Proteocephalidea): Compatibility of molecular and morphological data, and parasite-host coevolution. Int. J. Parasitol..

[37] Saitou N., Nei M. (1987). The neighbor-joining method: A new method for reconstructing phylogenetic trees. Mol. Biol. Evol..

[38] Tamura K., Stecher G., Kumar S. (2021). MEGA11: Molecular Evolutionary Genetics Analysis version 11. Mol. Biol. Evol..

[39] Suyapoh W., Sornying P., Thanomsub C., Kraonual K., Jantana K., Tangkawattana S. (2022). Distinctive location of piscine intestinal coccidiosis in Asian seabass fingerlings. Vet. World.

[40] Suyapoh W., Keawchana N., Sornying P., Tangkawattana S., Khirilak P., Jantrakajorn S. (2024). Mixed *Eimeria* and *Cryptosporidium* infection and its effects on pathology and clinical outcomes in juvenile Asian seabass (*Lates calcarifer*) cultured in Thailand. J. Fish Dis..

[41] Peng C., Tu G., Yu L., Wu P., Zhang X., Li Z., Li Z., Yu X. (2022). Murine chronic pancreatitis model induced by partial ligation of the pancreatic duct encapsulates the profile of macrophage in human chronic pancreatitis. Front. Immunol..

[42] Cuzzocrea S., McDonald M.C., Mazzon E., Siriwardena D., Calabrò G., Britti D., Mazzullo G., Caputi A.P., Thiemermann C. (2000). The tyrosine kinase inhibitor tyrphostin AG126 reduces the development of acute and chronic inflammation. Am. J. Pathol..

[43] Ondruschka B., Rosinsky F., Trauer H., Schneider E., Dreßler J., Franke H. (2018). Drug- and/or trauma-induced hyperthermia? Characterization of HSP70 and myoglobin expression. PLoS ONE.

[44] Jantrakajorn S., Keawchana N., Sornying P., Khirilak P., Suyapoh W. (2025). Intestinal inflammation, oxidative damage, and pathogenesis of intestinal *Cryptosporidium* in juvenile Asian sea bass (*Lates calcarifer*). J. World Aquac. Soc..

[45] Suyapoh W., Thaweechart B., Wae-Asae P., Keawchana N., Sornying P., Manmoo S., Rakwong P., Jantrakajorn S. (2025). Detection and pathological effects of intestinal parasites in spotted scat *Scatophagus argus*: *Filisoma* spp. and *Cryptosporidium* spp. as infective agents and their roles in fish inflammatory response. J. Aquat. Anim. Health.

[46] Sornying P., Keawchana N., Yakoh A., Nookamsuan K., Maitrimitr W., Sarawaree K., Jantrakajorn S., Suyapoh W. (2025). Piscine intestinal coccidial infections in pearl gentian groupers (*Epinephelus lanceolatus* ♂ × *E. fuscoguttatus* ♀): Pathogenesis and host intestinal response. Fish Pathol..

[47] Gibson D.I., Harris E.A., Bray R.A., Jepson P.D., Kuiken T., Baker J.R., Simpson V.R. (1998). A survey of the helminth parasites of cetaceans stranded on the coast of England and Wales during the period 1990–1994. J. Zool..

[48] Alvarado-Rybak M., Toro F., Abarca P., Paredes E., Español-Jiménez S., Seguel M. (2020). Pathological findings in cetaceans sporadically stranded along the Chilean coast. Front. Mar. Sci..

[49] Specht N.R., Keve G., Fernández-Maldonado C., Cerezo Caro A., Takács N., Kontschán J., Hornok S. (2024). Molecular investigation of endoparasites of marine mammals (Cetacea: Mysticeti, Odontoceti) in the Western Mediterranean. Front. Vet. Sci..

[50] Reckendorf A., Ludes-Wehrmeister E., Wohlsein P., Tiedemann R., Siebert U., Lehnert K. (2018). First record of *Halocercus* sp. (Pseudaliidae) lungworm infections in two stranded neonatal orcas (*Orcinus orca*). Parasitology.

[51] Cruz J.T., Ramilo D.W., Correia J., Afonso F., Pereira da Fonseca I., Cardoso L., Marzal A., Sequeira M., Falcão A., Madeira de Carvalho L. (2025). First report of three different parasite species in two dolphin species stranded on the coast of Portugal. Int. J. Parasitol. Parasites Wildl..

[52] Pool R., Fernández M., Chandradeva N., Raga J.A., Aznar F.J. (2020). The taxonomic status of *Skrjabinalius guevarai* Gallego & Selva, 1979 (Nematoda: Pseudaliidae) and the synonymy of *Skrjabinalius* Delyamure, 1942 and *Halocercus* Baylis & Daubney, 1925. Syst. Parasitol..

[53] Balseiro A., Herrero-García G., Royo L.J., Armenteros J.Á., Altonaga J.R., Monasterio J.M., Balsera R., Pool R.V., García Marín J.F., Pis-Millán J.A. (2023). Hypertrophic osteopathy in a common dolphin (*Delphinus delphis*) with concurrent pulmonary *Halocercus delphini* infestation. Heliyon.

[54] Pool R.V., Pons-García N., Consoli F., Rivero M., Bombardi C., Raga J.A., Aznar F.J. (2024). Fine-scale distribution of the lungworm *Halocercus delphini* in the lungs of the striped dolphin, *Stenella coeruleoalba*: Implications about migration pathways and functional significance. Mar. Mammal Sci..

[55] St. Leger J., Raverty S., Mena A., Terio K.A., McAloose D., St. Leger J. (2018). Cetacea. Pathology of Wildlife and Zoo Animals.

[56] Maganira J.D. (2026). *Stilesia hepatica* in African livestock: A review of pathology, liver condemnation, and economic losses. Prev. Vet. Med. Reg. Stud. Rep..

[57] Patten P.K., Rich L.J., Zaks K., Blauvelt M. (2013). Cestode infection in 2 dogs: Cytologic findings in liver and a mesenteric lymph node. Vet. Clin. Pathol..

[58] Guth C., Schumacher P.P., Vijayakumar A., Borgmann H., Balles H., Koschel M., Risch F., Lenz B., Hoerauf A., Hübner M.P. (2024). Eosinophils are an endogenous source of interleukin-4 during filarial infections and contribute to the development of an optimal T helper 2 response. J. Innate Immun..

[59] Yeshi K., Ruscher R., Loukas A., Wangchuk P. (2022). Immunomodulatory and biological properties of helminth-derived small molecules: Potential applications in diagnostics and therapeutics. Front. Parasitol..

[60] Muro A., Pérez-Arellano J.L. (2010). Nitric oxide and respiratory helminthic diseases. J. Biomed. Biotechnol..

[61] Pawłowska M., Mila-Kierzenkowska C., Szczegielniak J., Woźniak A. (2024). Oxidative stress in parasitic diseases—Reactive oxygen species as mediators of interactions between the host and the parasites. Antioxidants.

[62] Abou-El-Naga I.F. (2020). Heat shock protein 70 (Hsp70) in *Schistosoma mansoni* and its role in decreased adult worm sensitivity to praziquantel. Parasitology.

[63] Maresca B., Kobayashi G.S. (1994). Hsp70 in parasites: As an inducible protective protein and as an antigen. Experientia.

[64] Zakeri A. (2017). Helminth-induced apoptosis: A silent strategy for immunosuppression. Parasitology.

